# An Explainable Intelligence Driven Query Prioritization Using Balanced Decision Tree Approach for Multi-Level Psychological Disorders Assessment

**DOI:** 10.3389/fpubh.2021.795007

**Published:** 2021-12-17

**Authors:** Sushruta Mishra, Hrudaya Kumar Tripathy, Hiren Kumar Thakkar, Deepak Garg, Ketan Kotecha, Sharnil Pandya

**Affiliations:** ^1^School of Computer Engineering, Kalinga Institute of Industrial Technology, Deemed to Be University, Bhubaneswar, India; ^2^Department of Computer Engineering, Marwadi Unversity, Rajkot, India; ^3^Department of Computer Science and Engineering, School of Engineering and Sciences, Bennett University, Greater Noida, India; ^4^Symbiosis Centre for Applied Artificial Intelligence, Symbosis International (Deemed) University, Pune, India; ^5^Symbiosis Institute of Technology, Symbosis International (Deemed) University, Pune, India

**Keywords:** psychological risks, explainable intelligence, decision tree, oversampling, predictive learning

## Abstract

Human emotions affect psychological health to a great level. Positive emotions relate to health improvement; whereas negative emotions may aggravate psychological disorders such as anxiety, stress, and depression. Although there exist several computational methods to predict psychological disorders, most of them provide a black-box view of uncertainty. This research involves developing a novel predictive model for multi class psychological risk recognition with an accurate explainable interface. Standard questionnaires are utilized as data set and a new approach called a Q-Prioritization is employed to drop insignificant questions from the data set. Moreover, a novel balanced decision tree method based on repetitive oversampling is applied for the training and testing of the model. Predictive nature along with its contributing factors are interpreted with three techniques such as permuted feature importance, contrastive explanation, and counterfactual method, which together form a reasoning engine. The prediction outcome generated an impressive performance with an aggregated accuracy of 98.25%. The mean precision, recall, and F-score metric recorded were 0.98, 0.977, and 0.979, respectively. Also, it was noted that without applying Q-Prioritization, the accuracy significantly drops to 90.25%. The error rate observed with our model was only 0.026. The proposed multi-level psychological disorder predictive model can successfully serve as an assistive deployment for medical experts in the effective treatment of mental health.

## 1. Introduction

According to the observation of WHO, a healthy mind with physical well-being is possessed by a healthy individual (1). Also, it has been noted that various mental risks are gradually prevailing the society as a whole. In this fast-paced world with constant changes in the style of living, various kinds of psychological disorders are emerging in modern society. Mental health concerns including anxiety, stress, and depression are common in people's life. Both personal and professional instabilities contribute to the occurrence of these disorders. Also, it is noted that such psychological concerns exhibit many overlapping symptoms. As an instance, individuals experience loneliness in all the three mentioned disorders (2). These health risks are vital parameters of psychological well-being and must be given proper attention at right time in order to prevent any potential negative impact on individuals (3). Usually, experts take the help of questionnaires and interactive sessions to assess these mental risks issues. As per their observation, psychologically affected patients reserve their sentiments within themselves and are reluctant to share them with family, friends, or even medical professionals. Common symptoms observed in an anxious person include nervous feeling, frequent irritation, insomnia accompanied by fatigue, panic tendency, fast breathing, rapid pulse rate, and concentration issues (4). Similarly being regularly upset, unable to relax, less energetic, having chronic headaches, and overreacting to situations are some primary symptoms noted in stress disorder (5). Similarly, depression exhibits some vital factors like lack of focus, memory fluctuations, poor decision making, lack of zeal in recreation, feeling of helplessness and restlessness, weight loss, and suicidal mindset (6). Thus, as it is discussed, many common overlapping symptoms are observed in these three psychological risks like pain in the chest, insomnia, fatigue, pulse rate rise, and lack of concentration. The presence of multiple overlapping symptoms makes effective categorization of these disorders a concern. The gradual rise in these psychological risks has led clinical researchers to focus their explorations in this domain. Accurate assessment, categorization, and treatment of anxiety, stress, and depression disorders are also a challenging task for machines (7). So a suitable learning model is needed for reliable assessment and diagnosis (8). Magnificent success in data analytics has led to numerous applications of artificial intelligence. Continuous rise in these techniques leads to developing intelligent models with self-ability to perceive, learn, and take a decision on its own (9). But the efficiency and robustness of these autonomous models are restricted by their incapability to interpret their outcomes and decisive functionalities to users. The real concern is on relying on decisions that lack proper justification, thereby unable to provide a detailed explanation of certain system behaviors (10). These predictive models provide a black-box view which is tough to explain. However, with the rapid rise in deploying these self-learning models in various application domains, the need for transparency and interpretability is highly required from all stakeholders in data analytics (11). Especially in critical health related applications it requires an extensive explanation of any decision to medical experts rather than a simple projection of output for diagnosis. Thus, in recent times with the modern adoption of complex learning models in the healthcare field, more attention is to develop human interpretability enabled frameworks that can enhance its implementation capability. Thus, a new approach called explainable intelligence needs to be adopted that are modeled to use predictive learning methods that perform the following functionalities.

- Generate explainable frameworks maintaining a high level of accuracy.
- Enabling end users to trust and understand the underlying functional characteristics.
- Ensuring impartial decision making.
- Determining all relevant factors which are essential for the prediction of an outcome.

Predictive models with such explainable intelligence capability can readily provide explanation toward any outcome, weighs pros and cons of a model and, thereby suggest on the future behavior of the developed model. These interpretability based frameworks can be integrated with system interfacing to transform a predictive decision into an upgraded reasoning prototype with relevant explanation to users.

**Figure 1:**
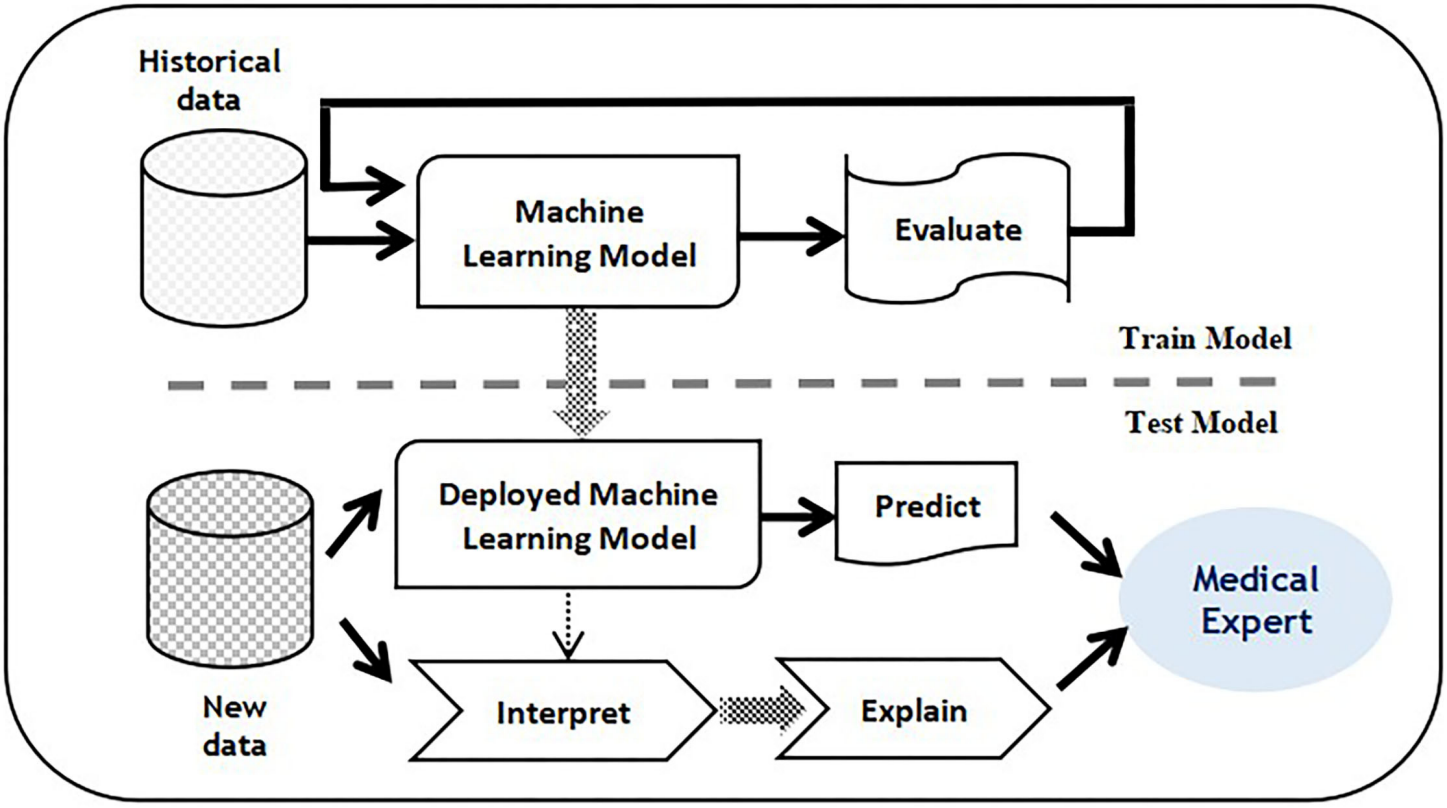
Training and testing phase with proposed Balanced decision tree approach.

The purpose of implementing explainable intelligent techniques is to appropriately explain the interpretation of predictive decisions obtained. Both the machine learning unit and the explainable intelligent unit are supplied with data and through the use of an explainable unit, precise explanations combined with prediction decisions are generated. These explanations can assist healthcare professionals to verify the decision of the predictive unit. Furthermore, it can be utilized with medical reports to provide in-depth reasoning and recommendations. A sample illustration of a predictive model supported by explainable intelligence is shown in Figure 1. Explainable intelligence is characterized by some vital elements which distinguish it from other conventional learning models. These critical elements concerned with an explainable intelligent model are discussed in this study.

- **Trust** is denoted by the certainty of a predictive model to perform during any troubleshooting.
- **Causality** is determined by the easiness to find associations such that there exists a strong logical link among variables.
- **Transferable** is governed by the interpretation of internal links occurring in a model which helps the user in reusing information to solve different problems.
- **Informative** property is based on the need for enormous information to reason predictive outcomes of a model, thereby avoiding local minima.
- **Fairness** symbolizes clarity of visualized results and the ethical ability to identify factors that affect a specific outcome.
- **Accessible** represents a feature allowing users to involve in the procedure of enhancing and building a predictive framework. Interactive is the capability of a learning model to share data and interact with the end user for effective interpretation.
- **Privacy** explores the functionality to interpret the internal links of a learning model by another third party to compromise the security aspects of the information.

## 2. Problem Statement

Psychological disorder risks such as anxiety, stress, and depression are quite common in modern society. There exist several prediction models that use different algorithms to detect the presence of these mental health issues at an early stage. Unfortunately, these standard machine learning techniques present a black box view whose predictive nature cannot be easily interpreted by medical experts. With the rise of computationally intelligent models in the critical healthcare sector, effective reasoning and transparent functionalities are very essential since a contradictory predictive result may affect the mental stability of a patient. Therefore, a trade-off must be addressed between the prediction and transparent capability for critical use cases that almost benefit more reliably and implements a robust system to assist medical experts to find it easy to backtrack a certain psychological risk recognition. Thus, the high predictive ability should be well supported by an explainable interface to be more productive. In this study, we present an explainable intelligence-enabled psychological disorders predictive model comprising a transparent and reliable intelligent model to detect acute mental disorders from accumulated online questionnaires through interactive sessions with patients.

## 3. Related Works

Various academicians and researchers throughout the world have undertaken numerous quality oriented works related to healthcare prediction (12, 13) across different domains (14, 15) using recent technologies. Researchers have also analyzed psychological health disorders using many predictive learning models. Different datasets are used for this purpose. Evaluation of several existing models are also done to figure out the best performing models. But no model is singled out to deliver the optimum performance. Based on varying use cases, scenarios, dataset composition, training algorithm employed, and other miscellaneous factors, the performance fluctuates. Some high profile works which are significantly suited for the domain are discussed in this section. Dooshima et al. (16) have utilized learning models using several characteristic features such as biological, geographical, mental risks, and environment based variables to predict psychological disorders in patients. Many health professionals verified the authenticity of the model along with these metrics. Srividya et al. (17) framed a questionnaire to retrieve data of various features which was later used to detect mental risks in people. Among machine learning algorithms used in the study, nearest neighbor and random forest gave identical and good results. Dabek and Caban (7) applied an artificial neural network model for forecasting mental health ambiguities which include anxiousness and depression tendency. The study also stressed the impact of injuries and concussions on the health of sportspersons. A detailed analysis concerning various predictive models such as support vector machine, neural network, and k-nearest neighbor was undertaken for psychological patients by Alonso et al. (18). The review concluded that using information mining methods on depression and dementia-like disorders can yield fruitful outcomes and can enhance the life style of patients (19). Sau and Bhakta (20) applied several data analytics algorithms such as regression, boosting, and Naive Bayes to classify patients affected with mental disorders. In this study, around 500 data samples were collected through interviews, and it was observed that boosting algorithm gave an optimal precision and accuracy value of 84.1 and 82.6%, respectively. A joint modeling architecture was developed by Saha et al. (21) where many linguistic variables on online sites were selected to classify psychological issues of virtual communities. The model performed better than other task learners, and the result explored the presence of sentiments beyond depression in the majority of online patients. Reece et al. (22) discussed the prediction parameters of depression and stress among the Twitter community where stress level was gauged through Hidden Markov Model (HMM). It was noted that around 24% and 32% of people were diagnosed with stress and depression tendency, respectively. Braithwaite et al. (23) used a decision tree model to determine patients with suicidal mindsets. About 135 tweets of different candidates were aggregated from Amazon Mechanical Turk and an accuracy rate of 92% was recorded. Du et al. (24) fetched Twitter samples with psychiatric stress units to tag suicidal tweets. Among all learning methods used, it was noted that convolution neural network performed the best among others including support vector machine and extra trees giving a 78% precision value in identifying online tweets with a suicide mindset. Al Hanai et al. (25) proposed a text-audio framework to detect depression where long short-term memory neural network was utilized to classify patients suffering from depression. The context-free method generated optimal results for multi-modal audio analysis. Ramiandrisoa et al. (26) predicted depression during early phases using social media data. CLEF eRisk tool was used to collect data. After an extensive evaluation, it was inferred that integration of information retrieval and machine learning produced the best outcome. Big data analytics was applied by Hou et al. (27) to forecast frustration and depression tendencies on basis of the reading pattern of users. Variables of Chinese textual data were retrieved to build a meta classification model. Naive Bayes gave the best performance among all. Leightley et al. (28) detected traumatic-related stress risks in ex-serviceman militants with supervised intelligent classifiers using various metrics such as gender, job state, and alcohol usage among others. It was observed that a good sensitivity rate was obtained for many classifiers except for the false negatives outcome. Young et al. (29) scanned facial expressions and body language of patients to detect mood variation and anxiety risks. The cross-validation method was used to generate a better precision rate and was alter validated by statistical algorithms. Mary et al. (30) conducted an experimental validation of stress, anxiety, and depression assessment with multiple machine intelligence methods using the DASS 21 dataset. Logistic regression gave the best performance with an accuracy of 90.33% on both stress and depression while 92% on anxiety risks. A detailed survey with around 6,000 candidates was done by Kessler et al. (31) to assess the degree of depression through the information mining approach and traditional methods. It was discovered that machine learners outperformed the traditional approach in detecting depression accurately. De Beurs et al. (32) presented an expert system with intervention mapping to help enhance user involvement in activities. The work discussed various patient specific design strategies for mental medicare using limited resources. Peng et al. (33) used kernel of support vector machine with some other learning classifiers to categorize depression patients on virtual community-based Twitter data. An accuracy rate of 83.46% was noted with kernel-based method. Anupriya et al. (34) analyzed various degrees of stress, depression, and anxiety risks using machine learning methods through DASS 2 questionnaires. Small dataset size was the major limitation of this study. Hatton et al. (35) assessed persistent depression in senior citizens through the gradient boosting method. In this study, PHQ-9 questionnaire was used for data collection purposes. Many such relevant existing research works are summarized in Table 1. Moreover, in Ray et al. (47), assessment of autistic disorder using machine learning approach is described. In Tripathy et al. (48), application and evaluation of classification model to detect autistic spectrum disorders in children are described. In Ray et al. (49), a review on Facial Expression Based Behavioral Analysis Using Computational Technique for Autistic Disorder Patients is described. In Mishra and Mohanty (50), an integration of machine learning and IoT for assisting medical experts in brain tumor diagnosis is described.

**Table 1:** Relevant works on psychological risk analysis using predictive analytics.

**References**	**Reviewed risks**	**Approach**	**Inference**
Bauer et al. (36)	Bipolar disorder	Paper-based survey	47% elderly people utilized internet and 87% youths exhibit bipolar disorder.
Dhaka and Johari (37)	Mental disorder	Genetic algorithm and MongoDB tool	Storage and processing massive mental risks data on MongoDB database.
Kumar and Bala (38)	Depression	Sentimental analysis and save data on Hadoop	Preprocessing online social media perspective on specific business products.
Furnham (39)	Personality disorder	Hogan “dark side” measure (HDS) concept of dependent personality disorder (DPD)	Most personality risk factors are highly linked to a type of cooperative personality.
Bleidorn and Hopwood (40)	Personality assessment	Prediction models and K-fold validation	Focused on aspects such as organized adaptability and arguments to improve verification of predictive techniques.
Sarraf and Tofighi (41)	Alzheimer's risk	Convolutional neural network	Mental health instances were successfully categorized with 96.86% accuracy rate.
Fiscon et al. (42)	Brain disorders	Decision tree and EEG signals	Decision tree outperforms others in precise risk detection with 90% accuracy and 87% specificity with use of cross validation method.
Chatterjee et al. (4)	Anxiety analysis	Regression and bayesian classifiers	Used a probabilistic technique to validate patients with anxiety levels. It concluded that Bayesian Network showed the best accuracy of 73.33%.
Omurca and Ekinci (43)	Traumatic stress risks	Neural networks and social media optimization	A hybrid system to classify PTSD individuals and allowed feature selection methods to find vital metrics of patients' risks. The accuracy differed between 74 and 79%.
Dabek and Caban (7)	Mental risks	Neural network	Analyzed 89,840 samples and recorded a classification accuracy of a range (73%-95%).
Katsis et al. (44)	Anxiety disorders	Integrated meta classifiers	Proposed a hybrid model with mental health signals for assessing anxiety risks. Accuracy of 77.33, 80.83, and 78.5% was the output with neural network, radial networks, and SVM, respectively.
Saxe et al. (45)	Stress risks	SVM and Lasso regression	Optimal AUC value noted was 79 and 78% with SVM and RF, respectively.
Karstoft et al. (46)	Stress and depression	Hybrid method Feature selection and SVM	Target Information Equivalence Algorithm optimized detection of PTSD when used with support vector machine. The mean AUC was 0.75.

All these discussed work models provide good prediction and classification of psychological health risks. Various machine learning and attribute optimization methods are applied for precise analysis of such mental health concerns in humans. The majority of works used standard questionnaires based data set gathered through interactive face-to-face sessions. Also, the existing models have experimented on patients with varying age groups. Though all of these above mentioned works generate good performance, still they suffer from certain constraints. Problems related to the small sample size are a challenge for several deployed models. Another problem is the fact that data samples collected from interviews or manual queries based analysis restricts users from freely responding to any queries. As earlier observed, an individual having anxiety, stress, or depression symptoms are quite reserved within themselves rather than interacting with family, friends, or clinical staffs. They usually share their mind through anonymous platforms. But most importantly the biggest challenge lies in the fact that most of these predictive intelligent models simply provide black-box predictions without any reliable justified explanations of those mental health risk predictions. As a result, it creates ambiguities, and appropriate interpretations of a certain risk prediction can not be reasoned out. Thus, large sized dataset collected from online platforms are more desirable, and an explanatory interface is the need of the hour to properly interpret the psychological health prediction outcomes.

## 4. Proposed Methodology

The prime focus of the research is on recognizing physiological disorders such as anxiety, stress, and depression implementing an explainable intelligence based predictive model. Psychological disorder data samples in the form of responses of questionnaires are collected from the desired participants through Google forms and are stored for analysis. The proposed framework can be viewed to comprise certain interlinked functional phases which are discussed in this section.

1. Transform-Encode Phase.
2. Segregate-Label Phase.
3. Q-Prioritization Phase.
4. Train-Test Phase.
5. Prediction Interpret Phase.
6. Validate Phase.

The sequential six phases are required for a comprehensive psychological assessment. The Transform-Encode Phase is employed as the data samples are gathered in the form of the google form, the raw and unprocessed data need to be presented in a suitable template that can be easily interpreted. The Segregate-Label Phase is employed to partition the attributes of the data set into three distinct sets of physiological disorders such as anxiety, depression, and stress. After the data samples are pre-processed and labeled, it is followed by the Q-Prioritization phase which is responsible for dropping out less significant queries or attributes from the data set. Once the irrelevant attributes in the data set are eliminated, Train-Test Phase is used to learn through examples using an appropriate machine intelligence algorithm. The Prediction Interpret Phase provides a suitable explanation of the prediction outcomes.

### 4.1. Transform-Encode Phase

The retrieved data samples are gathered in the form of google responses from users. These raw unprocessed data need to be presented in a suitable template that can be easily interpreted. Thus, the collected responses are mapped onto a two-dimensional tabular structure by allowing it to be saved in a csv (comma separated values) file in a new editable excel sheet. If an online survey is followed then the Typeform tool can be applied to fetch the responses in the .csv file directly. If any other ways to fetch data in the spreadsheet is used then excel can be used in saving it as a .csv file. The collected data samples can be loaded and their attributes are summarized in the python platform with appropriate libraries in store. It is important to detect any missing values in the data as it affects the processing. Any data entries which are out of their predefined range are tagged as missing values. These values are counted, identified, and are accordingly replaced with the data value which is most common for that specific column. It is followed by the encoding of data where based on severity level, the responses are assigned numerical values in the range of 1 to 4. Finally, the rate point is determined on a [0,3] scale by subtracting 1 from the encoded value (51, 52).

### 4.2. Segregate-Label Phase

In this phase, the attributes of the dataset are partitioned into three distinct sets of physiological disorders which include anxiety, depression, and stress based on its belonging value defined at prior. Five severity levels of these disorders are considered and domain range for every level is set at prior corresponding to individual sets of anxiety, depression, and stress disorders. Normal (N), Mild (M), Moderate (MD), Severe (S), and Extremely Severe (ES) are the detected levels for the research work undertaken. The Weight Score (WS) for all disorder sets is calculated by summing up every data rate points for individual set of disorders taking into consideration of all column cell values and is given in Equation (1).


(1) $$\begin{array}{r} W_{S}=\sum RP^{i} \end{array}$$

In this study, *RP*_*i*_ denotes the rate points of an individual set of mental disorders. “ES” label for anxiety, stress, and depression risks was assigned a WS of 20+, 33+, and 28+, respectively. “S” severity levels for these three classes were allotted a WS range of 15–19, 26–33, and 21–27, respectively. Similarly “MD” labeled instances were adjusted WS range of 10–14, 19–25, and 14–20, respectively. The WS values for the “M” class for all the risks were 8–9, 15–18, and 10–13, respectively. The WS values less than the above range are considered to be the “N” class.

A sample data-sheet illustrating the query set corresponding to anxiety, stress, and depression risks is represented in Table 2. It shows the computation of WS for all tuples and based on which the severity class label is assigned.

**Table 2:** A sample weight score computation illustration.

**Q2**	**Q4**	**Q7**	**Q9**	**Q15**	**Q19**	**Q20**	**Q23**	**Q25**	**Q28**	**Q30**	**Q36**	**Q40**	**Q41**	**W_*s*_**	**Class**
**Anxiety**															
3	3	1	0	1	1	0	1	0	2	3	1	0	0	16	S
2	3	3	1	3	2	3	3	3	3	3	1	1	2	33	ES
3	0	0	0	0	0	1	0	2	0	1	2	0	0	9	M
0	2	0	0	0	0	1	0	0	0	1	1	0	1	6	N
1	0	2	1	0	1	2	0	1	0	0	1	2	0	11	MD
**Q1**	**Q6**	**Q8**	**Q11**	**Q12**	**Q14**	**Q18**	**Q22**	**Q27**	**Q29**	**Q32**	**Q33**	**Q35**	**Q39**	**W_*s*_**	**Class**
**Stress**															
2	1	3	2	3	3	3	3	3	2	3	3	3	2	36	ES
3	2	2	3	3	1	1	3	0	1	1	0	1	2	23	MD
3	2	3	0	3	3	0	2	3	1	3	3	3	2	31	S
2	2	0	0	1	2	0	0	0	0	1	0	0	0	8	N
2	0	0	2	0	0	0	2	0	3	2	3	1	1	16	M
**Q3**	**Q5**	**Q10**	**Q13**	**Q16**	**Q17**	**Q21**	**Q24**	**Q26**	**Q31**	**Q34**	**Q37**	**Q38**	**Q42**	**W**_*s*_	**Class**
**Depression**															
3	3	3	3	2	2	1	3	1	3	3	3	0	3	33	ES
0	1	2	1	0	2	1	1	0	0	0	2	1	0	11	M
1	0	0	0	1	0	0	0	0	1	0	0	0	1	4	N
1	2	1	0	0	0	3	3	1	3	0	2	3	3	22	S
3	1	2	0	0	1	0	1	2	2	2	1	1	2	18	MD

### 4.3. Q-Prioritization Phase

After the data samples are pre-processed and labeled, it is followed by the Q-Prioritization phase which is responsible for dropping out less significant queries or attributes from the dataset. It is to note that, the pre-processed question set is the input while a reduced and optimized question set is the output of the Q-prioritization phase. Sometimes it is observed that all attributes in the samples do not equally contribute to the processing and prediction process. The presence of these less relevant attributes affects the overall performance of the predictive model in the context of latency delay or accuracy of prediction. Thus, detecting and eliminating these less relevant features is a vital task. In this phase, a novel attribute relevance method is adopted before the model is subjected to training and testing using classifiers. The WS computed in the predecessor phase is analyzed and a relevant threshold (Rth) for every physiological disorder set is determined by Equation (2) as follows.


(2) $$\begin{array}{r} R_{th}=\frac{Normal_{max\left(PD\right)}}{Count_{col}\times Rate_{max}} \end{array}$$

A *R*_*th*_ for every physiological disorder set is determined in Equation (2). In this study, *Normal*_*max*(*PD*)_ represents the maximum value corresponding to the *N* level of any disorder set. The *Count*_*col*_ denotes the number of attribute columns present in the questionnaire data set, and *Rate*_*max*_ represents the optimum scaled rate point considered. Based on the equation, the *R*_*th*_ for every attribute column of the respective disorder set is computed. The computed *R*_*th*_ value for anxiety, stress, and depression is 0.5, 1.0, and 0.2, respectively. Cumulative summation of all rate points which are grouped by individual column name is found out and a simple mean average for every column is determined. Furthermore, the priority of attribute columns is calculated on the basis of the ranking of their mean value in descending order which is calculated in terms of priority relevance (*P*^*rel*^) as shown in Equation (3):


(3) $$\begin{array}{r} P^{rel}=\overset{S}{\underset{i=1}{\sum }}\frac{R_{i}}{S} \end{array}$$

In this study *P*^*rel*^ is the priority relevance value to be computed. *R*_*i*_ denotes the rate point for every data sample for a specific disorder set, and “S” is the total samples of data collected. Based on the computation of *P*^*rel*^ obtained, those attributes are considered to be relevant and are retained. The overall pseudo code of this procedure is depicted in Algorithm 1.

**Algorithm 1:** Pseudocode for Q-prioritization phase.

**Input**: *W*_*s*_
**Output**: Relevant prioritized queries.
**1** Scan all labeled *W*_*s*_ determined in “Segregate-weight score phase” ;
**2** Compute relevant threshold *R*_*th*_ for each *PD* subset: $R_{th}=\frac{Normal_{max\left(PD\right)}}{Count_{col}\times Rate_{max}}$ ;
**3 foreach** *disorder set* *D* **do** Add all rate points *R*_*i*_ grouped by column. ;
**4** Find mean μ_*i*_ for each column. ;
**5** Designate μ_*i*_ as *P*^*rel*^ ;
**6** Rank all *P*^*rel*^ in ascending order priority: $P^{rel}=\overset{S}{\underset{i=1}{\sum }}\frac{R_{i}}{S}$ ;
**7 if** $P^{rel}\leq R_{th}$ **then**
**8** | corresponding attribute column is irrelevant. ;
**9 end**
**10 if** $P^{rel}\geq R_{th}$ **then**
**11** | corresponding column is relevant. ;
**12 end**
**13** Relevant prioritized queries are retained. ;

### 4.4. Train-Test Phase

Once the irrelevant attributes in the dataset are eliminated, it is all set to learn through examples using an appropriate machine intelligence algorithm. After the model is adequately trained, it can be used to predict outcomes based on new data samples. In our research, an improved adaptation of the decision tree algorithm is deployed as the machine learning method. The decision tree is a widely used supervised approach to solve classification problems. It can be viewed as a hierarchical structure with internal nodes mapped onto attributes, branches denoting rules, and leaf nodes are the decision outputs. The decision node and leaf node are the two nodes of a decision tree. Decision nodes are responsible to take decisions while the outcome of decisions is denoted by leaf nodes. Based on the attributes of a dataset, decisions are made.


(4) $$\begin{array}{r} Gain\left(I,F\right)=Ent\left(I\right)-\underset{s\in values\left(F\right)}{\sum }\frac{\left|I_{s}\right|}{\left|I\right|}\times Ent\left(I_{s}\right) \end{array}$$

A decision tree works with the purpose of predicting the label of a data sample initialized from the root node of the tree. Values of the root node are compared to the data variable and on basis of comparison follow the branch and move to the subsequent node. The process further compares the feature against all sub-nodes and proceeds. The same procedure is continued till the leaf node is reached. Algorithm 2 relates its overall pseudocode.

**Algorithm 2:**
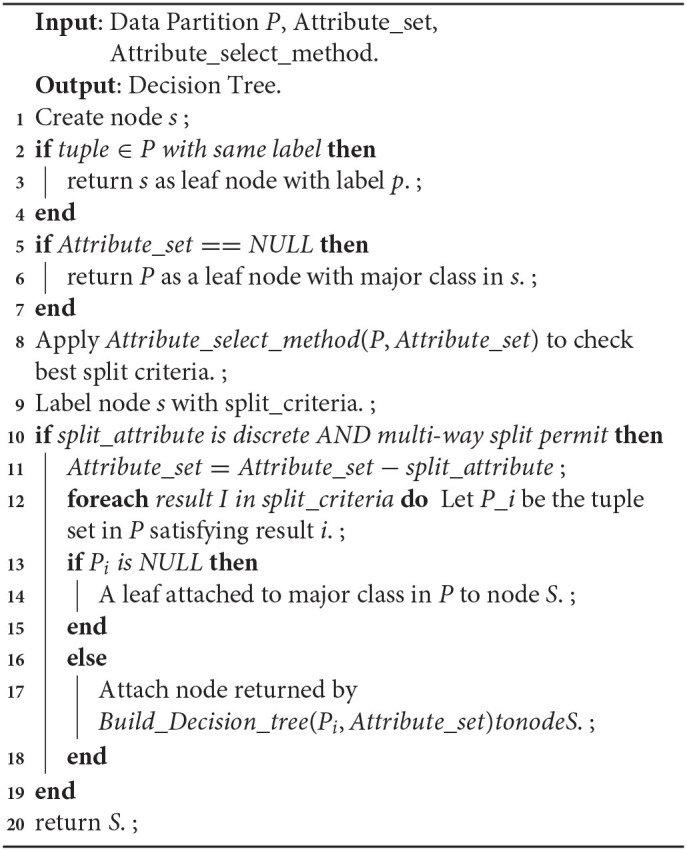
Decision tree building from training instances of data partition *P*.

One of the major concerns in a decision tree implementation is to choose the best attribute during the split. A suitable attribute selection measure solves the purpose. Information gain is a statistic based method for the selection of suitable attributes for testing at every node. It is a measure of entropy changes after attribute driven data segmentation which computes the information that an attribute gives regarding a class. Based on its value, the node is split, and accordingly, the decision tree is built. Gain (I; F) of an attribute A, that concerns a set of samples S is highlighted in Equation (4).

Where values (F) is the set of all possible values for attribute F, and Is is the subset of I for which the attribute F has value s. The pseudocode for information gain is shown in Algorithm 3. Entropy in information theory is a metric that exhibits the purity of a random dataset. When the target feature accepts m distinct values, then its entropy I corresponding to m-wise categorization is denoted by Equation 5.


(5) $$\begin{array}{r} Ent\left(I\right)=\overset{m}{\underset{n=1}{\sum }}-P_{i}\times log_{2}P_{n} \end{array}$$

where “Ent” represents the entropy and pi denotes the probability of I which belongs to a class i.e., the logarithm of base 2 refers to the entropy of encoded size determined in bits.

**Algorithm 3:**
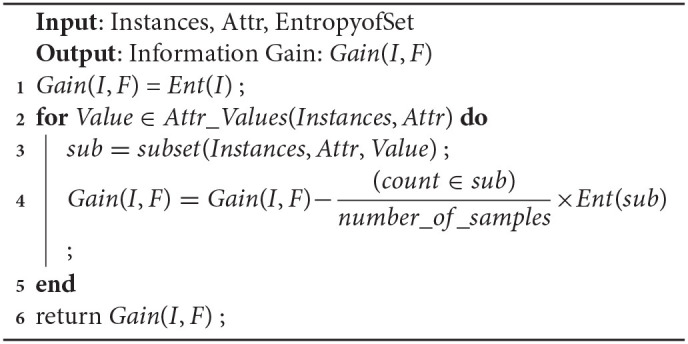
Pseudocode for Information gain.

#### 4.4.1. Proposed Balanced Decision Tree Method

It is observed that the majority of predictive learning techniques generate optimistic classification performance which is misleading at times due to the uneven distribution of data samples in classes. It is due to the fact that many computational algorithms are built to function on classification samples with identical observations in every class. Data sampling can be used to deal with this issue a sit constitutes a set of methods to map an unbalanced data set onto more evenly distributed classes. The decision tree is biased toward the majority class, and as a result, the mis-classification rate of the minor class is more. In this study, sampling process can be fruitful in tackling this uneven data distribution. One such type called oversampling process replicates existing samples from minor classes and augment those to the train set. Here, training samples are randomly chosen with replacement such that a more balanced data set can be created. A balanced decision tree model differs from a simple decision tree in that it incorporates the oversampling technique to create an even distribution of data samples in both major and minor classes. The performance of a balanced decision tree is much better than the simple decision tree.

The improved decision tree presented in this study utilizes the oversampling method to generate a more efficient model. The physiological disorder data set is pre-processed and less relevant attributes are removed using the attribute relevance phase. The entire dataset is then partitioned into anxiety, stress, and depression sets on the basis of the predefined attribute set. The data instances are mapped onto the five disorder levels using simple if-then rules. Based on the cumulative rate point of a specific row, that instance falls in its corresponding disorder level. The labeling step is followed by oversampling where the minority class is identified in the training samples and random replicas of its instances are created. The sampling rate “S” chosen is 0.1. A decision tree algorithm is applied to the resultant oversampled dataset, and the accuracy rate is computed. The initial accuracy before the first iteration is assumed to be zero (Aold = 0). If the recent computed accuracy is found to be more than the predecessor value then the latest value is stored, thereby incrementing the sampling rate by 0.1. Again the data is oversampled with S = 0.1 to find the new accuracy. The process is repeated as long as the new accuracy Anew for a round is less than its previous value. At that point, the algorithm stops and generates the maximum accuracy value obtained throughout the process and is restored. After the training of the decision tree is done, the new test data is subjected to the model to predict the level of physiological disorder in the patient.

**Figure 2:**
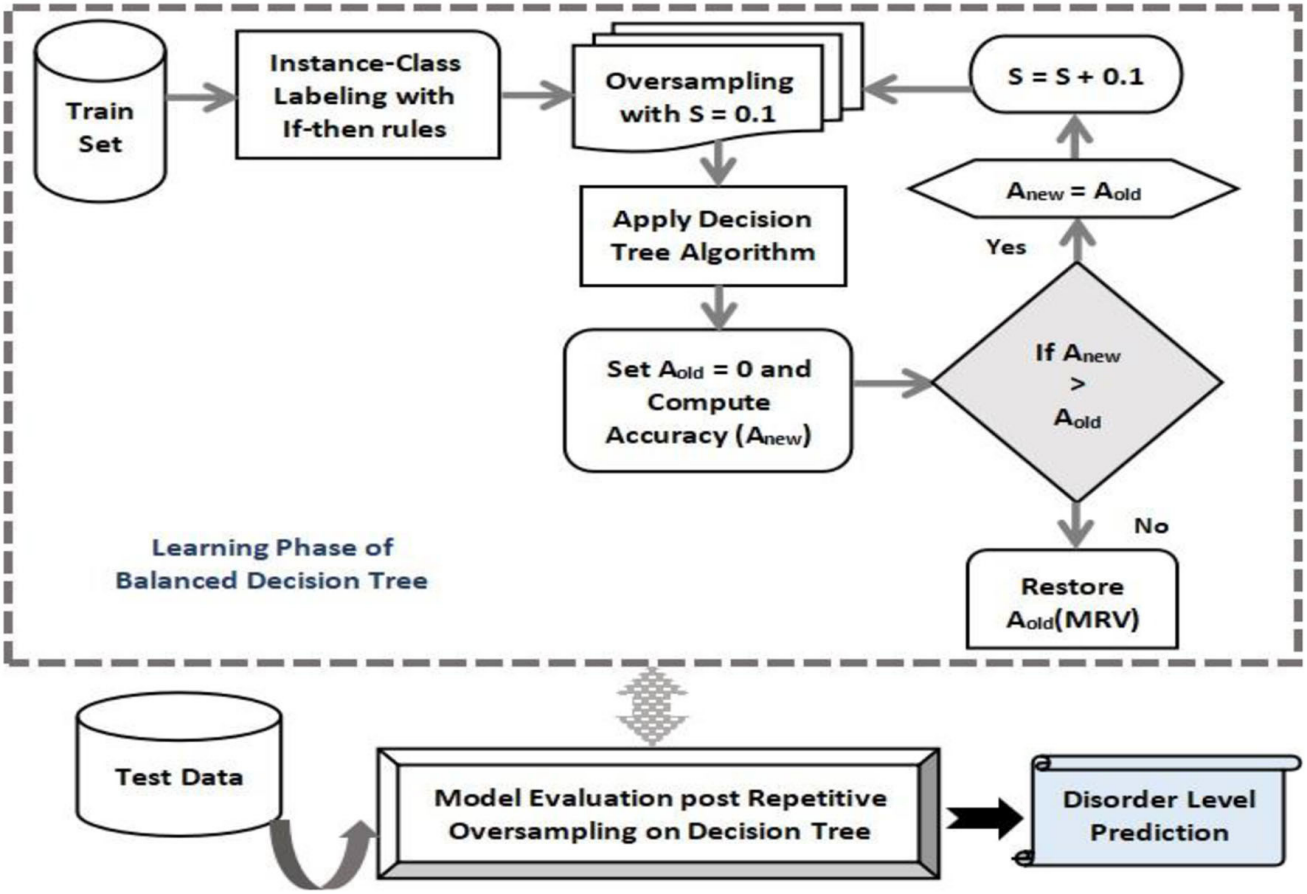
Proposed Balanced decision tree approach.

Thus, the developed model successfully detects the presence of any mental risk disorder, and if any inconsistencies are found then it predicts the level of disorder that the patient has probably developed corresponding to each set of a physiological disorder (anxiety, stress, and depression). Since the oversampling process is repeated multiple times in learning of decision tree to form a more evenly balanced data set, it is referred to as Balanced Decision Tree model which is illustrated in Figure 2.

### 4.5. Prediction Interpret Phase

An important observation that should be addressed is the set of attributes that have more impact on prediction outcomes. Most predictive models focus simply on the prediction performance rather than providing a suitable explanation for it. This creates an ambiguous scenario where factors that contributes to a certain prediction can not be determined. This creates a concern, especially when dealing with the real time critical healthcare domain. In our research analysis of detecting psychological health risk severity, an appropriate interpretation of predictive results is necessary from the medical experts and the patient's perspective. Thus, a predict interpret phase is augmented in our proposed model to facilitate the explainable intelligence functionality. This explainable intelligence capability is operated through a reasoning engine. The main aim of this reasoning engine is to allot a scoring value to all attributes on basis of their individual usefulness and relevance to a certain prediction. This module helps in providing a detailed insight in to the dataset. The scoring level of attributes can identify their relevance ranking which can be further used by medical experts to reason a particular mental health risk prediction. Our model implements a novel reasoning engine that consists of permuted feature importance, contrastive explanation, and counterfactuals methods for empowering the explainable intelligence functionality. The permutation feature importance explores the relationship between a specific attribute and the target label where a reduction in score indicates the dependency of that attribute on the developed model. The contrastive explanation method (CEM) aims to provide local interpretations to an abstract black box system that discusses explanation for classification by detecting less relevant features. A counterfactual interpretation of a prediction defines the variation in features that alters the prediction to a predetermined outcome.

#### 4.5.1. Permuted Feature Importance Method

Permuted feature importance is an approach to calculate the relative significance scores of attributes and is independent of the model used. It acts as a non-local interpretation approach providing an in-depth idea of the nature of a predictive learner. Attributes are ranked on basis of the significance of every attribute on the trained learning algorithm predictive decision. Feature permutation importance computes the prediction ability of an attribute for a black box determinator by exploring the increase in prediction error if an attribute is absent. It begins with the calculation of base error on a trained predictive model. For each attribute, random shuffling of the corresponding column of trained data is performed, and the predicted error of the varied samples is computed. Then the feature importance is determined and stored as the difference between the base score and the varied sampled score. These steps are repeated multiple times to determine the average as it reduces the impact of random shuffling. Subsequently, on basis of the mean relevance of attributes on the score of the model, their ranking is done. Attributes with high score relevance on shuffling are labeled as more significant compared to attributes with less impact on the score of the model. Its pseudocode is shown in Algorithm 4.

**Algorithm 4:**
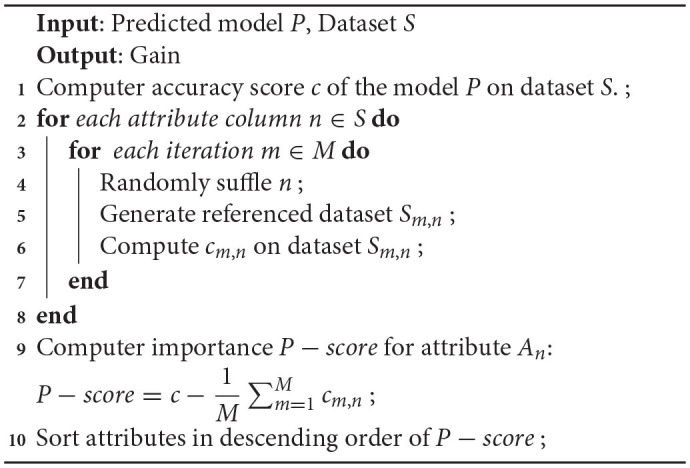
Pseudocode for permuted feature importance method.

Permutation feature selection can be used *via* the permutation_importance() function that takes a fit model, a dataset (train or test dataset is fine), and a scoring function P-score. The relative attribute importance can be illustrated in a bar chart where the x-axis shows the importance value and the y-axis denotes the attributes in significance order. A long bar is an indicator for an attribute with more relevance.

#### 4.5.2. Contrastive Explanation Method

**Figure 3:**
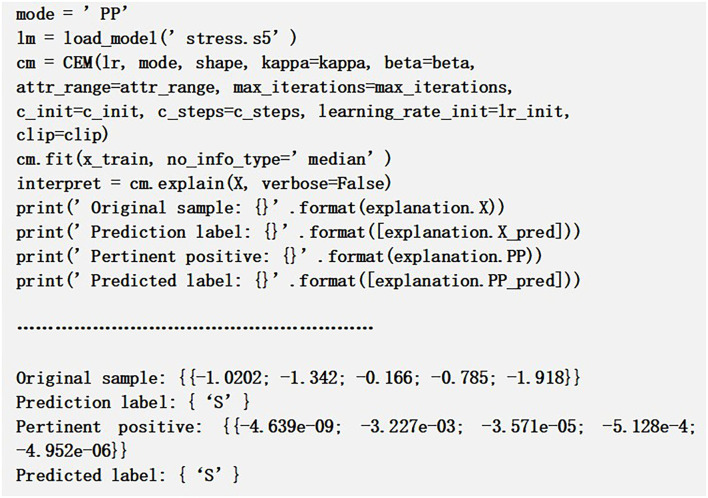
Pertinent positive sample example.

Contrastive explanation method is another explainable method that derives local interpretations of a black box architecture. Technically it may be defined as “An input m is categorized into class n since attributes ai,…aj are available and since attributes ax,…ay are unavailable.” This approach suits more for tabular sample data which explains a classification model by giving details on relevant attributes or pertinent positives (PP) as well as irrelevant attributes or pertinent negatives (PN). It is a process that reasons out what may be minimally present and which variables may be essentially absent from the samples so that the original predicted class can be maintained. The two interpretation sides can be denoted as follows:

*Pertinent Positives:* An explanation to the PP figures out the attributes that are vital for model to predict the same output class as the predicted class. So from our research context, it consists of the crucial queries of the psychological risk data that impact most on the prediction outcome. The above result in Figure 3 highlights the prediction of mental risk severity level is the same when PP is applied. The generated CEM values near 0 since those are the least values present to predict specific risk levels and these queries are to be essentially present to obtain the same original level as the severity level predicted S.

*Pertinent Negatives:* Explanation to PN computes the attributes which are to be adequately unavailable, form a sample, thereby retaining the original output level.

**Figure 4:**
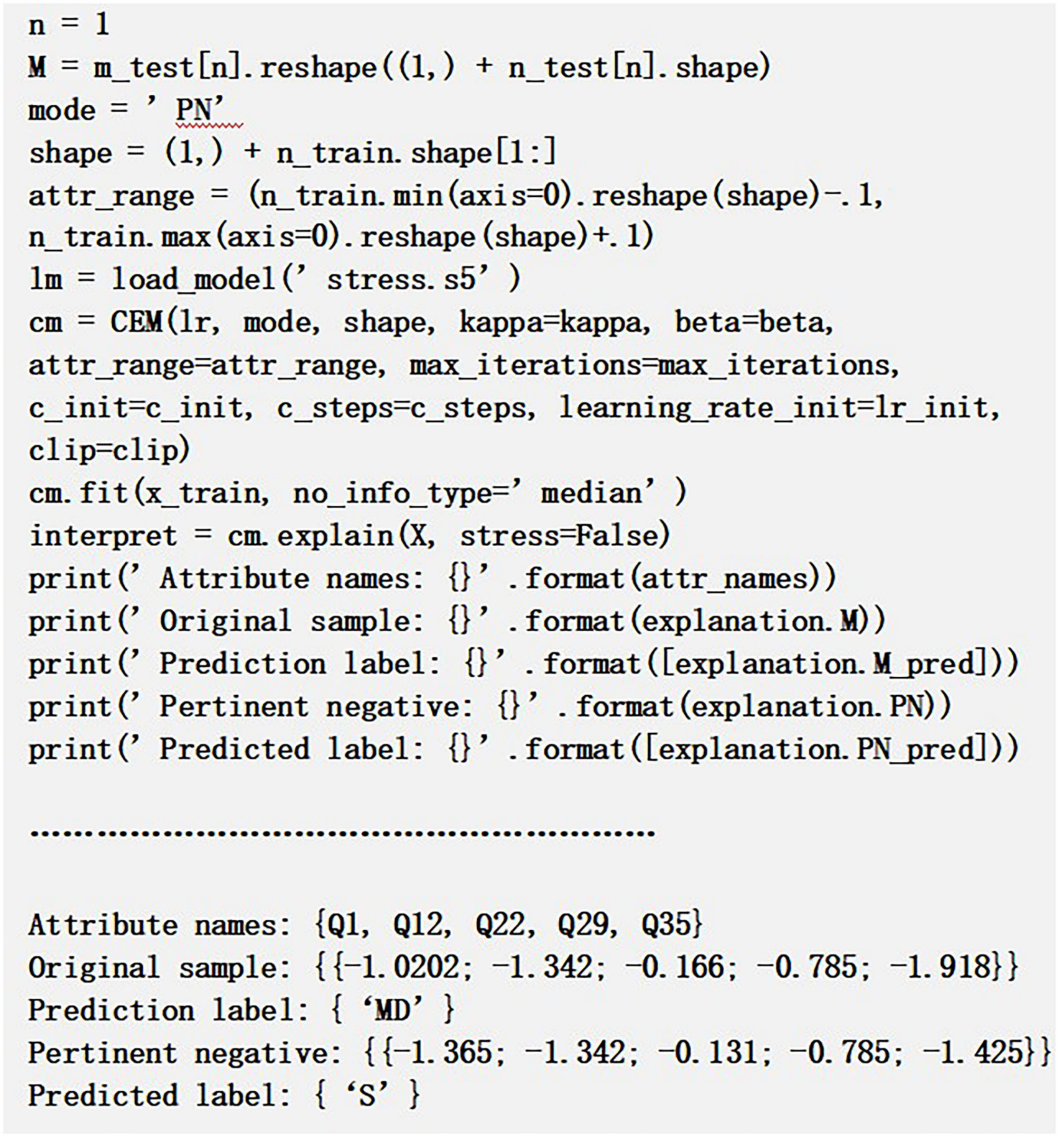
Pertinent negative sample example.

It can be seen from Figure 4 that some of the CEM values in the array such as Q1, Q3, and Q5 are different from the original values, and these values alter the prediction class from “MD” to “S.” Hence, we may conclude that variation in these attribute queries may essentially be absent in order to restore the original predicted class since they can flip the class label. Using CEM, we can improve the accuracy of the predictive model by analyzing the misclassified samples and subsequently processing those through CEM explanations.

#### 4.5.3. Counterfactuals Method

Counterfactuals method provides a somehow “what-if” explanations based on prototypes where an instance represent the whole sample set of a class. Technically, it can be said that if M and N are independent and dependent variables, respectively, then counterfactual depicts the impact on N as a result of minor variation in M. Thus, it notifies the variation to be performed on M so as to alter the output from N to N. It is fast and gives quite accurate results by interpreting results with the use of prototypes of the target class. In context to our research analysis, a counterfactual sample denotes the vital modification in input query set of a testing sample which change the prediction to a predetermined outcome. A counterfactual sample Sxy should possess some basic characteristics which are outlined in this study.

1. The prediction over *S*_*xy*_ should be enclosed in a predetermined output label.
2. Variation β of the initial sample *S*_0_ over *S*_*xy*_ = *S*_0_ + β needs to be sparse.
3. The counterfactual *S*_*xy*_ should be near to the general counterfactual class sample sharing.
4. The counterfactual *S*_*xy*_ should be computed fast to be applied in real time use cases.

These features can be approved by including a loss function in the form denoted by Equation 6 as:


(6) $$\begin{array}{r} Loss=xL_{Pred}+\beta L_{1}+L_{2} \end{array}$$

**Figure 5:**
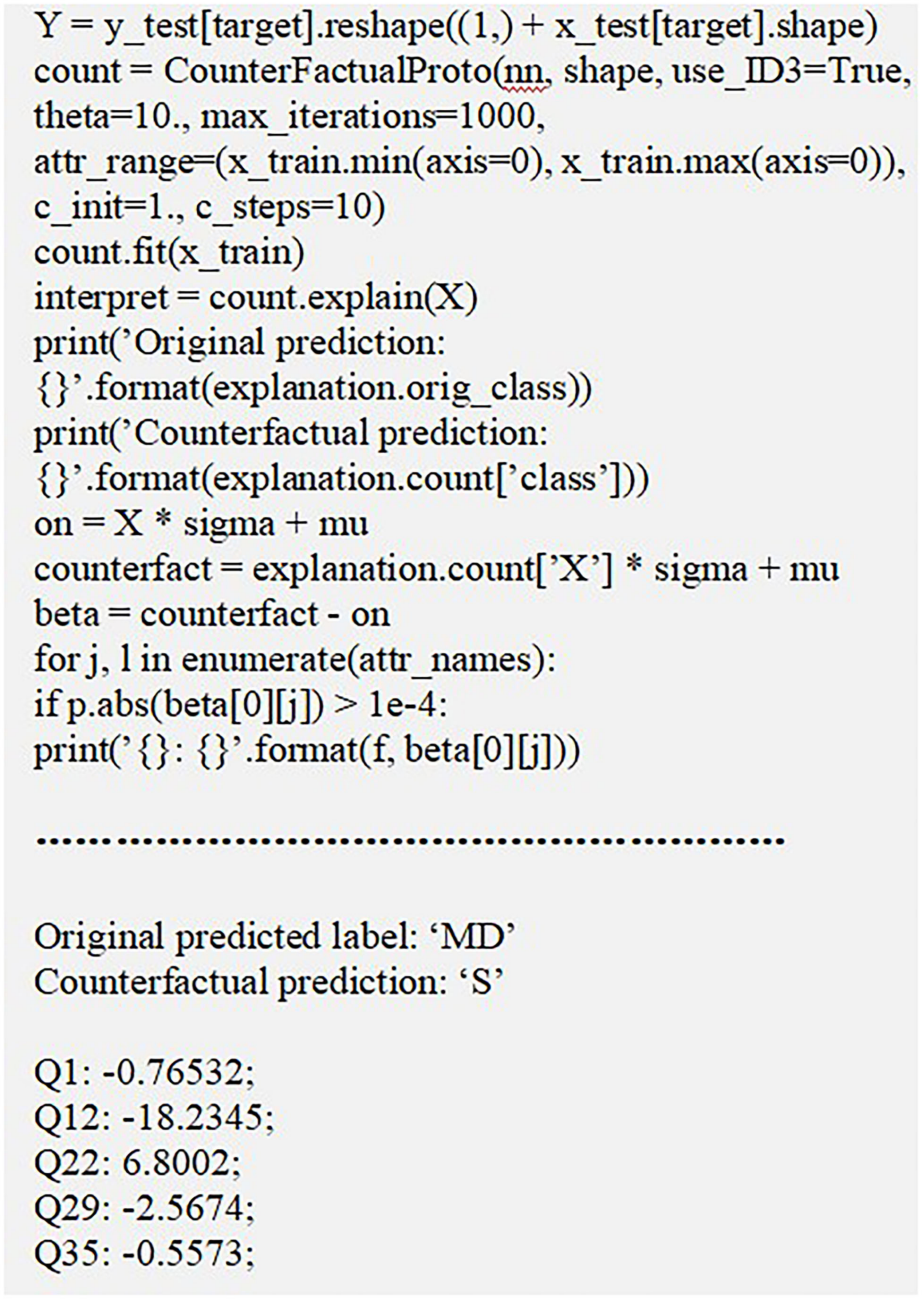
Sample demonstration of counterfactual method.

Where *xL*_*pred*_ denotes the instance variation for predicting another class label than the original class. β*L*_1_ + *L*_2_ acts as the regularizer that defines sparsity by considering the net difference between counterfactual and alternate samples. In this study, as shown in Figure 5, samples of mentally disordered person with anxiety were collected and diagnosed. After diagnosis and an evaluation prototype was created to detect variations in various attributes to analyse risk possibilities. The observation highlights certain modifications to alter the outcome. The query Q1 (being upset) should be decreased by 0.76532 to avoid getting “S” risk level. Q12 (being nervous) should be under control by 0.182345 units. Similarly, as noted in Q29(–2.5674) and Q35(–0.5573), the patient should calm down more and be more tolerant to prevent being in “S” level of risk.

#### 4.5.4. Validate Phase

Usually among all psychological health response data instances used as input, few among them may be misclassified to incorrect class in a predictive model. Thus, the performance of the proposed explainable intelligence enabled predictive model needs to be validated to figure out its effectiveness. Both training and testing of the model require validation using different metrics. Various performance parameters are involved in demonstrating its efficiency in detecting correct outcomes. Accuracy is a vital parameter that indicates the total mental health disorder instances that were correctly predicted among all the instances which were used as input to the predictive model. It is depicted in Equation 7 as:


(7) $$\begin{array}{r} Accuracy=\frac{TP+TN}{FP+FN} \end{array}$$

Precision denotes the number of accurate mental health risk instances predicted which in reality maps to be positive. It is a fruitful indicator where false positives is a concern than false negatives. It is shown in Equation 8 as:


(8) $$\begin{array}{r} Precision=\frac{TP}{TP+FP} \end{array}$$

The recall represents the actual positive risk instances that accurately predict the model, and it is a very helpful indicator where false negative exceeds false positives. It is represented in Equation 9 as:


(9) $$\begin{array}{r} Recall=\frac{TP}{TP+FN} \end{array}$$

In reality, if the precision is increased then the recall value decreases and vice-versa. To capture both metrics simultaneously, another measure called F-score is used to present a combined concept of both. It denotes the harmonic average of both recall and precision. The F-Score metric is denoted in the Equation 10 as:


(10) $$\begin{array}{r} F-Score=2\times \frac{Precision\times Recall}{Precision+Recall} \end{array}$$

Mean square error (MSE) is another relevant metric that accepts the mean value of the square of the difference of the original and the predictive values which are represented by Equation 11 as:


(11) $$\begin{array}{r} MSE=\frac{1}{M}\overset{M}{\underset{i=1}{\sum }}{\left(Z_{i}-\bar{Z_{i}}\right)}^{2} \end{array}$$

Where *M* represents the data points count, *Z*_*i*_ is the observed value, and $\bar{Z_{i}}$ is the predicted value.

**Figure 6:**
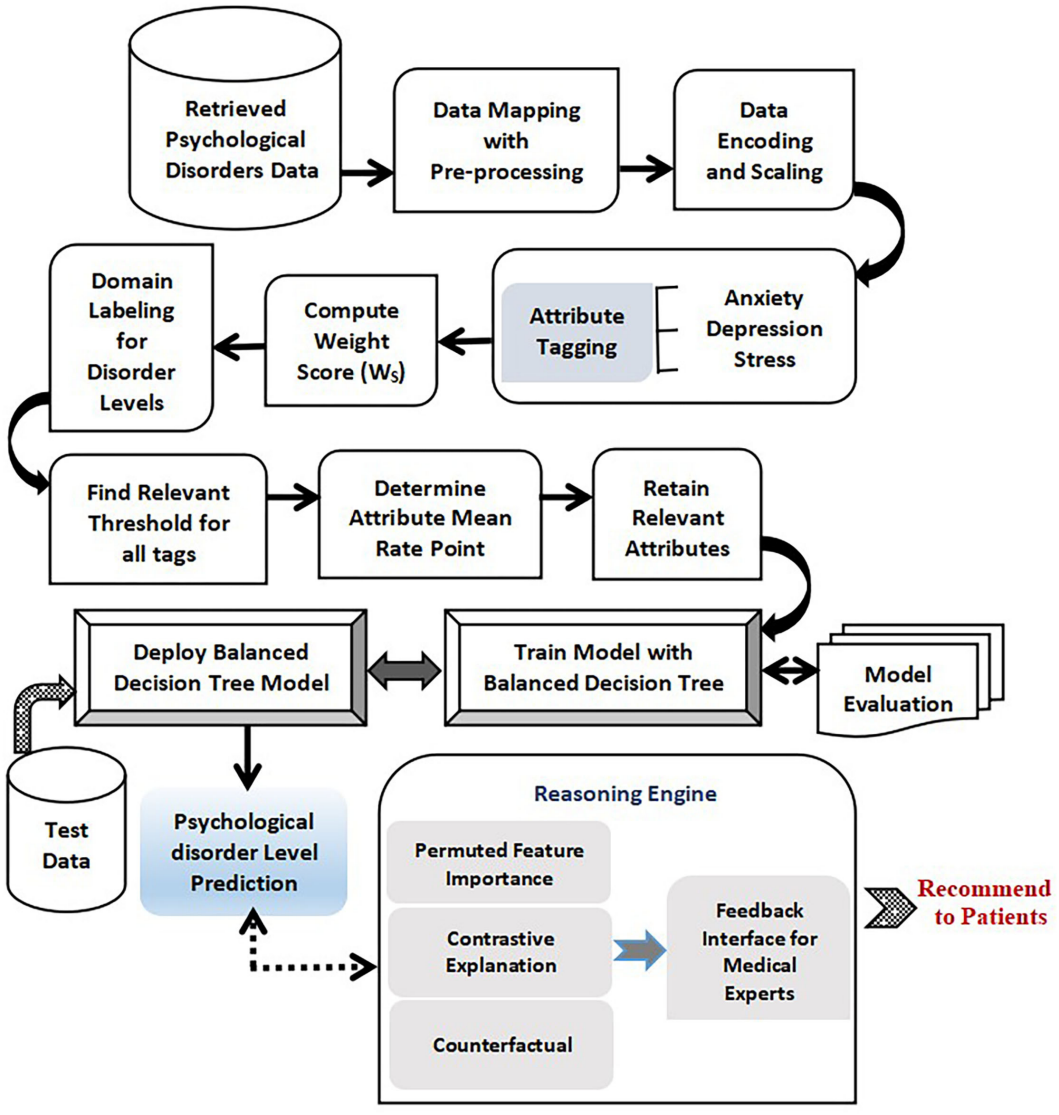
Proposed Explainable Intelligence driven psychological health predictive model.

Thus, as it is observed, all the phases of the proposed explainable intelligence model are interrelated such that the output of one phase acts as the input for the other. The overall working model is summarized in Figure 6. The psychological health risk data DASS 42 is retrieved online. The dataset is in the form of 42 questionnaires along with its responses. The original data samples are mapped onto an excel sheet for analysis. It undergoes appropriate pre-processing tasks like handling missing values and any inconsistencies in the dataset. The data is then encoded in the range of [1–4] based on the response of patients and scaled up to [0–3] for uniformity and ease of analysis. The dataset is grouped by predefined queries to partition into three subsets on the basis of psychological health disorders. Anxiety, stress, and depression are the identified subsets. The WS of all rows is determined, and accordingly, the risk severity level is labeled. The least priority queries are identified using the attribute mean rate point method and are eliminated from the dataset in Q-Prioritization phase. Then the resultant data is trained with a novel, newly proposed balanced decision tree technique which involves repeated oversampling. Once the model is trained, it can predict the appropriate disorder severity level for anxiety, stress, and depression risks with new unseen psychological data of new patients. The prediction outcome is further analyzed and interpreted using the reasoning engine which is comprised of three constituents. The first component called permuted feature importance method ranks all queries in priority order to identify the most relevant queries which contributed to prediction. The contrastive explanation approach further helps in recognizing the queries which should be present and similar queries that should be absent for the prediction of the severity risk level. Based on this, an interpreted tabular feedback is generated for reference of medical experts. The counterfactuals method is the third reasoning method that defines the required variations required in input queries in order to flip the generated severity class label. Thus, this reasoning engine forms the most vital part of the model a sit empowers the predictive model with an explainable intelligence capability.

## 5. Implementation Results and Analysis

The proposed explainable intelligence based mental health risk detection model is tested with the newly built balanced decision tree technique to predict the severity level of anxiety, stress, and depression health risks in patients. Before real time deployment, it is validated with different performance parameters to explore its efficiency in detecting psychological disorders.

### 5.1. Training Data Evaluation Performance

**Figure 7:**
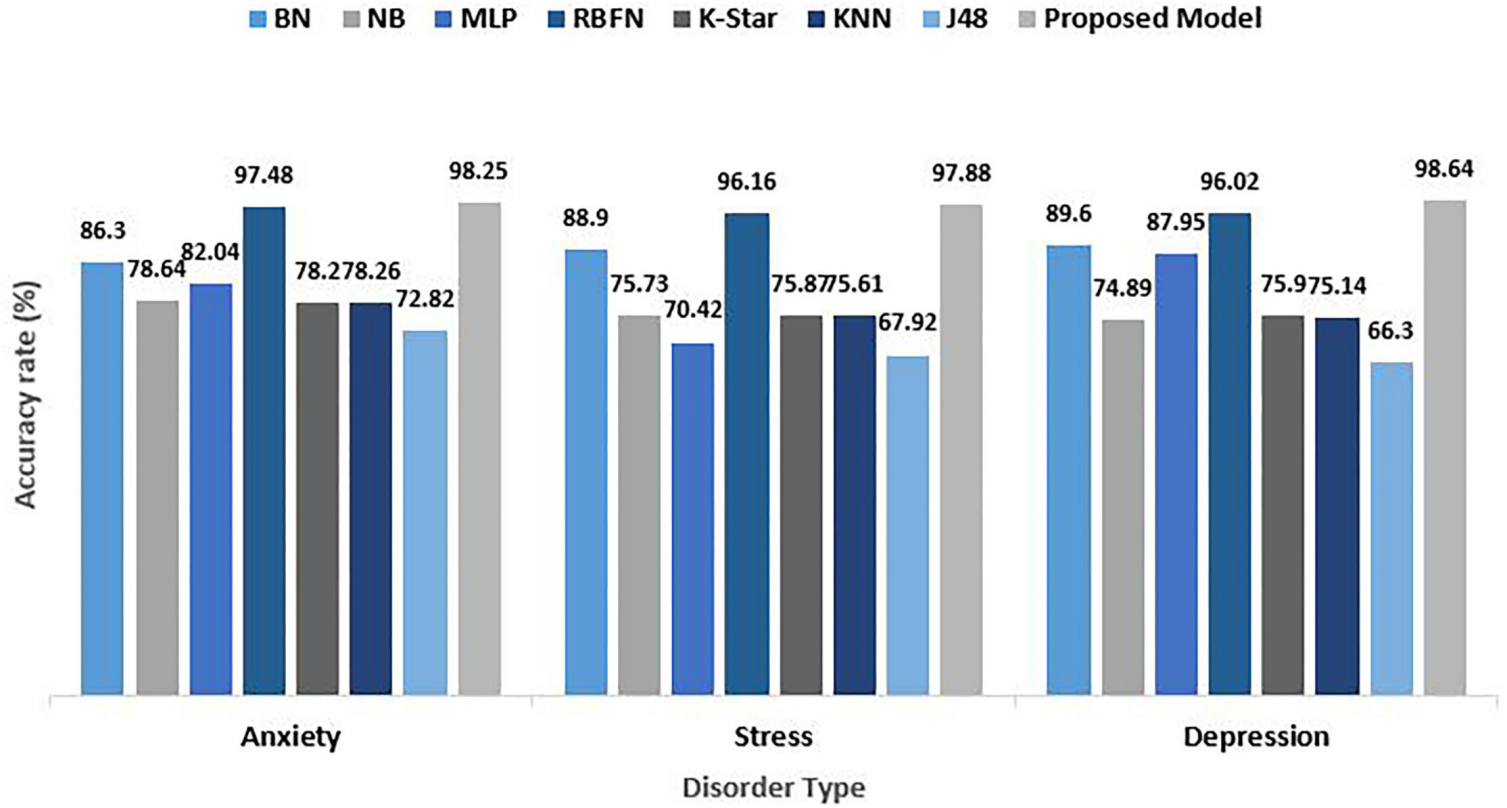
Comparative analysis of accuracy rate for psychological risks.

The psychological health disorder dataset used for performance evaluation is the standard DASS 42 questionnaires. As many as 13,000 responses of patients forms the dataset. With the help of the performance parameters indicated in the previous section, the performance is determined. The dataset is experimentally trained using many predictive models such as Naive Bayes, RBFN, KNN, J48, and neural network apart from the proposed model. Accuracy is the most important evaluation parameter which denotes the proportion of accurate predictions to the cumulative observations. Accuracy is a very relevant metric in an evenly balanced data samples. It is observed that among all existing techniques, the RBFN model provided an impressive accuracy on all three datasets while the accuracy of J48 was relatively discouraging. But our proposed explainable intelligence model outperformed others recording an accuracy rate of 98.25%, 97.88%, and 98.64% on anxiety, stress, and depression set, respectively, as shown in Figure 7. The aggregated accuracy computed was 98.25%.

**Figure 8:**
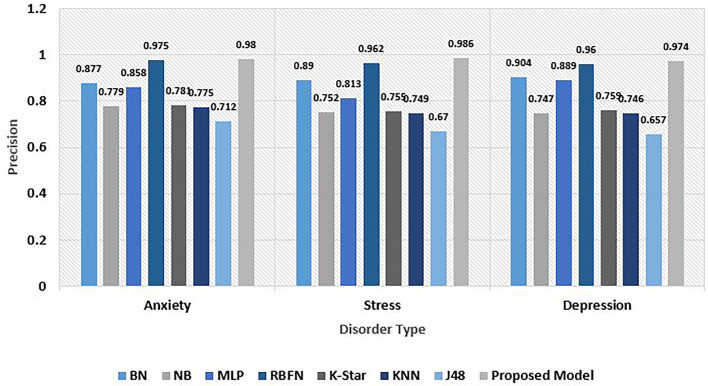
Comparative analysis of precision for psychological risks.

Precision represents the proportion of accurate positive prediction observations to the cumulative positive prediction based observations. Precision evaluates the accurately categorized samples against those samples classified to be positive. The model performed exceptionally well in terms of precision metrics on all three risks. A value of 0.98 was observed with anxiety data, 0.986 on stress data, and 0.974 on depression data. Thus, a simple mean value of 0.98 precision was noted leaving behind the RBFN model which has a 0.968 precision value. J48 performed relatively poor in comparison with others. Figure 8 depicts the precision score analysis of the proposed explainable model with others.

**Figure 9:**
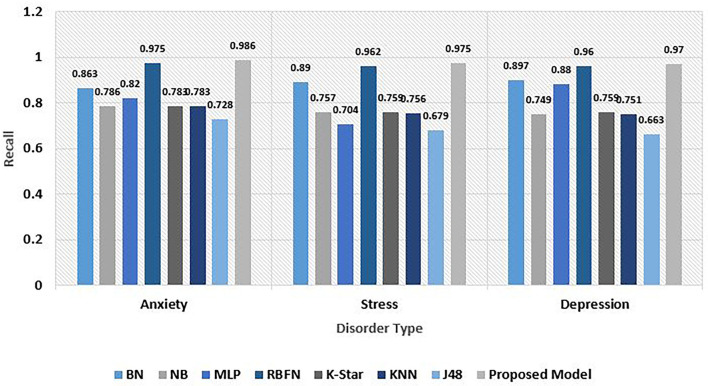
Comparative analysis of Recall for psychological risks.

Recall metric helps in the quantification of accurate positive predictions among all positive predictions performed. While precision gives an idea of accurate positive predictions, recall gives a measure of missed out positive predictions. For imbalanced learning, recall is typically used to measure the coverage of the minority class. The explainable driven predictive model outperforms all other existing models in terms of recall value on all disorder data. While an excellent value of 0.986 is the result with anxiety data, on stress and depression set, the recall metric evaluates to be 0.975 and 0.97, respectively. The aggregated recall observed was 0.977. The recall analysis is depicted in Figure 9.

**Figure 10:**
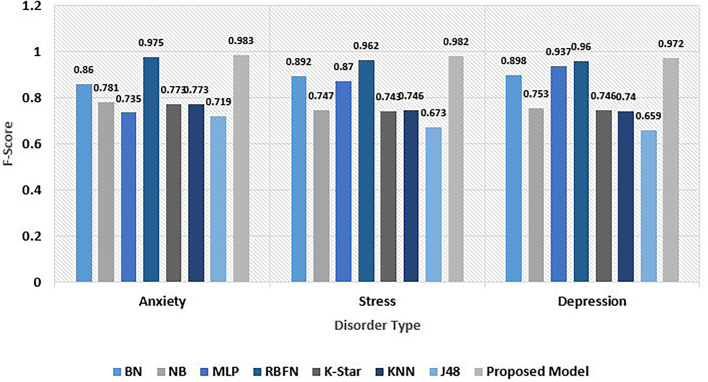
Comparative analysis of F-Score for psychological risks.

F-score is a means to integrate both recall and precision metrics, thereby capturing both features. Once both of the metrics are computed for our multi-level classification, an F-score can be utilized to combine both generating a harmonic average value. F-score considers both false negatives and false positives. The F-score value recorded on the anxiety, stress, and depression risk set was 0.983, 0.982, and 0.972. The average mean F-score value generated was 0.979. Among other models, the RBFN algorithm recorded a very good value while J48 gave a comparatively inferior outcome. The overall F-score analysis was observed in Figure 10.

**Figure 11:**
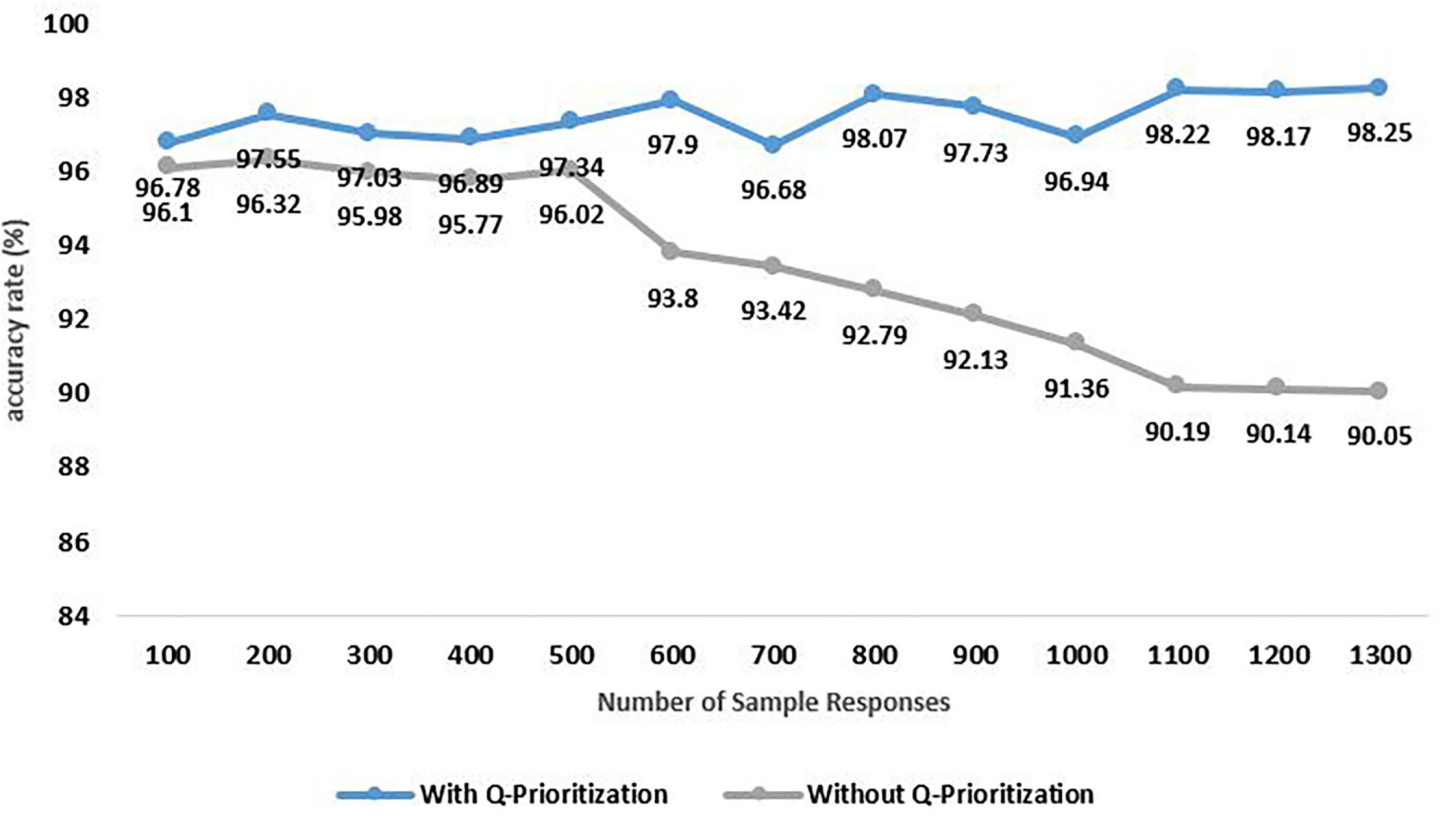
Accuracy rate analysis of psychological risks in context to Q-Prioritization phase.

The impact of the Q-Prioritization phase on the performance was also explored and the outcomes are illustrated in Figure 11. The observation was quite clear. Right from the initial data size of 100 instances, the accuracy of the explainable model using Q-Prioritization phase, 96.78% was more than that of the model deployed without it, 96.1%. As we can see that the accuracy shows a significant drop once the dataset size exceeds 500 instances. The difference in accuracy increases with the rise in the size of data and toward the end of simulation with 1,300 samples. The final accuracy without the Q-Prioritization phase is found to be 90.05%.

The experimental evaluation was also undertaken with DASS 21 data set that constitutes 21 queries being mapped onto anxiety, stress, and depression risk. The same performance indicators were used for the evaluation. We obtained 98.09, 0.971, 0.97, and 0.972% as the mean accuracy, precision, recall, and F-score value obtained with DASS 21 sample set. This shows the consistent performance of our proposed model with heterogeneous data sets. The outcome summary is outlined in Table 3. There is variation in values because two different data sets (DASS 42 and DASS 21) are used for the evaluation of the proposed research on mental risk disorders. Both these data sets comprise dissimilar questions. Also, distinct questions are associated with both data sets so based on the feature importance of individual questions, the values for accuracy and other parameters are different.

**Table 3:** Performance metrics comparison using different datasets.

	**DASS 42**	**DASS 21**	
Accuracy	0.982	98.16	Anxiety
Precision	0.98	0.976	
Recall	0.986	0.982	
F-Score	0.983	0.978	
Accuracy	97.88%	97.69%	Stress
Precision	0.986	0.98	
Recall	0.975	0.968	
F-Score	0.982	0.975	
Accuracy	98.64%	98.42%	Depression
Precision	0.974	0.958	
Recall	0.97	0.962	
F-Score	0.972	0.96	

### 5.2. Real Time Prediction Outcome

The experimental evaluation was undertaken in a real time environment. Our proposed model was validated with volunteers. DASS-42 is the standard dataset used for the purpose. The dataset constitutes a series of 42 distinct queries being asked to patients affected with psychological disorder risks. The queries were equally clustered into anxiety, stress, and depression which forms the three predefined sets as discussed earlier. So every risk set comprises 14 non-overlapping queries. Tables 4–6 displays the 14 queries each chosen for anxiety, stress, and depression disorder set.

**Table 4:** Queries associated with anxiety risk.

**Query**	**Description**
Q2	I was aware of dryness of my mouth.
Q4	I experienced breathing difficulty such as rapid breathing and breathlessness.
Q7	I had a feeling of shakiness such as legs going to give away.
Q9	I found myself in situations that made me so anxious I was most relieved when they ended.
Q15	I had a feeling of faintness.
Q19	I perspired noticeably in the absence of high temperatures of physical exertion.
Q20	I felt scared without any good reason.
Q23	I had difficulty in swallowing.
Q25	I was aware of the action of my heart in the absence of physical exertion.
Q28	I felt I was close to panic.
Q30	I feared that I would be thrown by some trivial but unfamiliar task.
Q36	I felt terrified.
Q40	I was worried about situation in which I might panic and make a fool of myself.
Q41	I experienced trembling in the hands.

**Table 5:** Queries associated with stress risk.

**Query**	**Description**
Q1	I found myself getting upset by quite trivial things.
Q6	I tended to over-react to situations.
Q8	I found it difficult to relax.
Q11	I found myself getting upset rather easily.
Q12	I felt that I was using a lot of nervous energy.
Q14	I found myself getting impatient when I was delayed in any way.
Q18	I felt that I was rather touchy.
Q22	I had it hard to wind down.
Q27	I found that I was very irritable.
Q29	I found it hard to calm down after something upset me.
Q32	I found it difficult to tolerate interruptions to what I was doing.
Q33	I was in a state of nervous tension.
Q35	I was intolerant of anything that kept me from getting on with what I was doing.
Q39	I found myself getting agitated.

**Table 6:** Queries associated with depression risk.

**Query**	**Description**
Q3	I could not seem to experience any positive feeling at all.
Q5	I just could not seem to get going.
Q10	I found that I had nothing to look forward to.
Q13	I feel sad and depressed.
Q16	I felt that I had lost interest in just about everything.
Q17	I felt I was not worth much as a person.
Q21	I felt that I was not worthwhile.
Q24	I could not seem to get any enjoyment out of the things I did.
Q26	I felt down-hearted and blue.
Q31	I was unable to become enthusiastic about anything.
Q34	I felt I was pretty worthless.
Q37	I could see nothing in the future to be hopeful about.
Q38	I felt that life was meaningless.
Q42	I found it difficult to work up the initiative to do things.

**Figure 12:**
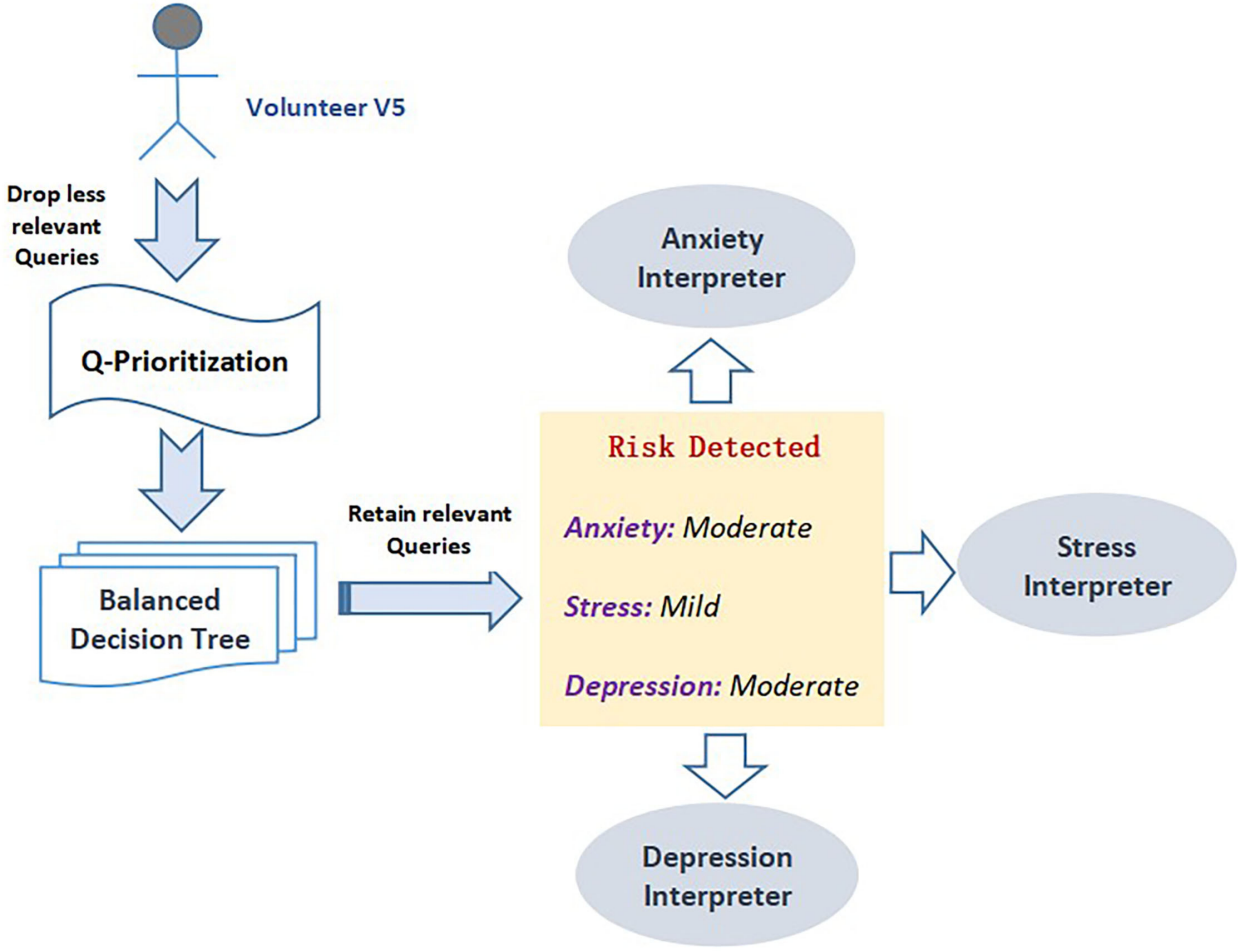
Demonstration of prediction interpret phase.

Figure 12 depicts the google form prepared for our research hypothesis. This form comprises a total of 42 distinct questionnaires which are mandatory to be filled up by users. This acts as a self-testing interface for the users in an attempt to test themselves of the presence of any psychological health risks and is called psychology parser. The user needs to enter his name and ID before appearing in the test.

The outcome demonstration for one specific day is discussed in this section where the test data of 10 volunteers are accumulated and their responses are mapped onto an excel sheet in a .csv file. It is followed by the important Q-Prioritization phase which is responsible for identifying less relevant queries from all sets and eliminating them using the mean Rate Point method. Table 7 demonstrates this process. It starts with the original query set segregated into 14 queries in each disorder set including anxiety, stress, and depression. The individual queries are assigned their scaled-up value (0–3) based on the response of patients. The priority relevance value (Prel) for all queries (attributes) corresponding to individual disorder sets are determined using the mean rate point method.

**Table 7:** Demonstration of Q-Prioritization phase.

	**Q2**	**Q4**	**Q7**	**Q9**	**Q15**	**Q19**	**Q20**	**Q23**	**Q25**	**Q28**	**Q30**	**Q36**	**Q40**	**Q41**
**Anxiety risk set**														
V1	3	3	1	0	1	1	0	1	0	2	3	1	0	0
V2	2	3	3	1	3	2	3	3	3	3	3	1	1	2
V3	3	0	0	0	0	0	1	0	2	0	1	2	0	0
V4	0	2	0	0	0	0	1	0	0	0	1	1	0	1
V5	1	0	2	1	0	1	2	0	1	0	0	1	2	0
V6	3	0	0	0	0	0	1	0	3	0	1	2	0	0
V7	0	2	0	0	0	0	1	0	0	0	0	1	0	1
V8	2	0	2	1	0	1	2	0	1	0	0	1	2	0
V9	3	3	1	0	1	2	0	1	0	2	3	1	0	0
V10	2	2	3	1	3	1	3	2	3	3	3	1	1	2
Mean	1.9	1.5	1.2	0.4	0.8	0.8	1.4	0.7	1.3	1.0	1.5	1.2	0.6	0.6
	**Q1**	**Q6**	**Q8**	**Q11**	**Q12**	**Q14**	**Q18**	**Q22**	**Q27**	**Q29**	**Q32**	**Q33**	**Q35**	**Q39**
**Stress risk set**														
V1	2	1	3	2	3	3	3	3	3	2	3	3	3	2
V2	3	2	2	3	3	1	1	3	0	1	1	0	1	2
V3	3	2	3	0	3	3	0	2	3	1	3	3	3	2
V4	2	2	0	0	1	2	0	0	0	0	1	0	0	0
V5	2	0	0	2	0	0	0	2	0	3	2	3	1	1
V6	2	3	0	0	1	2	0	0	0	0	1	0	0	1
V7	1	0	0	2	0	0	0	2	0	3	2	3	1	1
V8	3	2	3	0	3	3	0	2	3	1	3	3	3	1
V9	2	1	3	2	3	3	3	3	3	2	3	3	2	2
V10	3	2	2	3	3	1	1	3	0	1	1	0	1	1
Mean	2.3	1.5	1.6	1.4	2.0	1.8	0.8	2.0	1.2	1.4	2.0	1.8	1.5	1.3
	**Q3**	**Q5**	**Q10**	**Q13**	**Q16**	**Q17**	**Q21**	**Q24**	**Q26**	**Q31**	**Q34**	**Q37**	**Q38**	**Q42**
**Depression Risk set**														
V1	3	3	3	3	2	2	1	3	1	3	3	3	0	3
V2	0	1	2	1	0	2	1	1	0	0	0	2	1	0
V3	1	0	0	0	1	0	0	0	0	1	0	0	0	1
V4	1	2	1	0	0	0	3	3	1	3	0	2	3	3
V5	3	1	2	0	0	1	0	1	2	2	2	1	1	2
V6	1	3	1	0	0	0	3	3	1	3	0	2	3	3
V7	3	1	2	0	0	1	0	1	2	1	2	1	1	2
V8	1	1	0	0	0	0	0	0	0	1	0	0	0	1
V9	3	3	3	3	2	2	1	3	2	3	3	3	0	3
V10	0	1	2	1	0	2	1	1	0	0	0	3	1	0
Mean	1.6	1.6	1.6	0.8	0.5	1.0	1.0	1.6	0.9	1.7	1.0	1.7	1.0	1.8

This is followed by the ranking of the queries based on the *P*^*rel*^ value obtained. Accordingly, the least priority query that fails to satisfy the *R*_*th*_ is eliminated from the resultant data set. As it is observed in **Table 10**, query “Q9” was found to be less relevant in the anxiety set. Similarly, queries “Q18” and “Q5” in stress and depression set, respectively, were detected to be at less priority compared to others. Thus, these least priority queries were dropped from the data set after the Q-Prioritized phase. The least priority queries are marked in bold in Table 8.

**Table 8:** Irrelevant queries detection and elimination in Q-Prioritization phase.

**Anxiety**														
Q2	Q4	Q7	Q9	Q15	Q19	Q20	Q23	Q25	Q28	Q30	Q36	Q40	Q41	Pre-Q-Prioritized
Q9	Q40	Q41	Q23	Q15	Q19	Q28	Q7	Q36	Q25	Q20	Q4	Q30	Q2	Post-Q-Prioritized
**Stress**														
Q1	Q6	Q8	Q11	Q12	Q14	Q18	Q22	Q27	Q29	Q32	Q33	Q35	Q39	Pre-Q-Prioritized
Q18	Q27	Q39	Q11	Q29	Q6	Q35	Q8	Q14	Q33	Q12	Q22	Q32	Q1	Post-Q-Prioritized
**Depression**														
Q3	Q5	Q10	Q13	Q16	Q17	Q21	Q24	Q26	Q31	Q34	Q37	Q38	Q42	Pre-Q-Prioritized
Q5	Q13	Q26	Q17	Q21	Q34	Q38	Q3	Q5	Q10	Q24	Q31	Q37	Q42	Post-Q-Prioritized

The psychological parser is definitely technically feasible. It is a kind of google form software created at the back-end of model development in which all questions related to the research task is included and randomly shuffled. Patients are required to fill up the parser and then the risks of anxiety, stress, and depression are auto-computed. So it is completely software enabled with no complex hardware involved.

Once the data set undergoes the Q-Prioritized phase, it is pre-processed and is ready for prediction. Test data sample of any particular volunteer is subjected to the proposed Balanced Decision Tree algorithm model for detection of psychological disorder risks.

**Figure 13:**
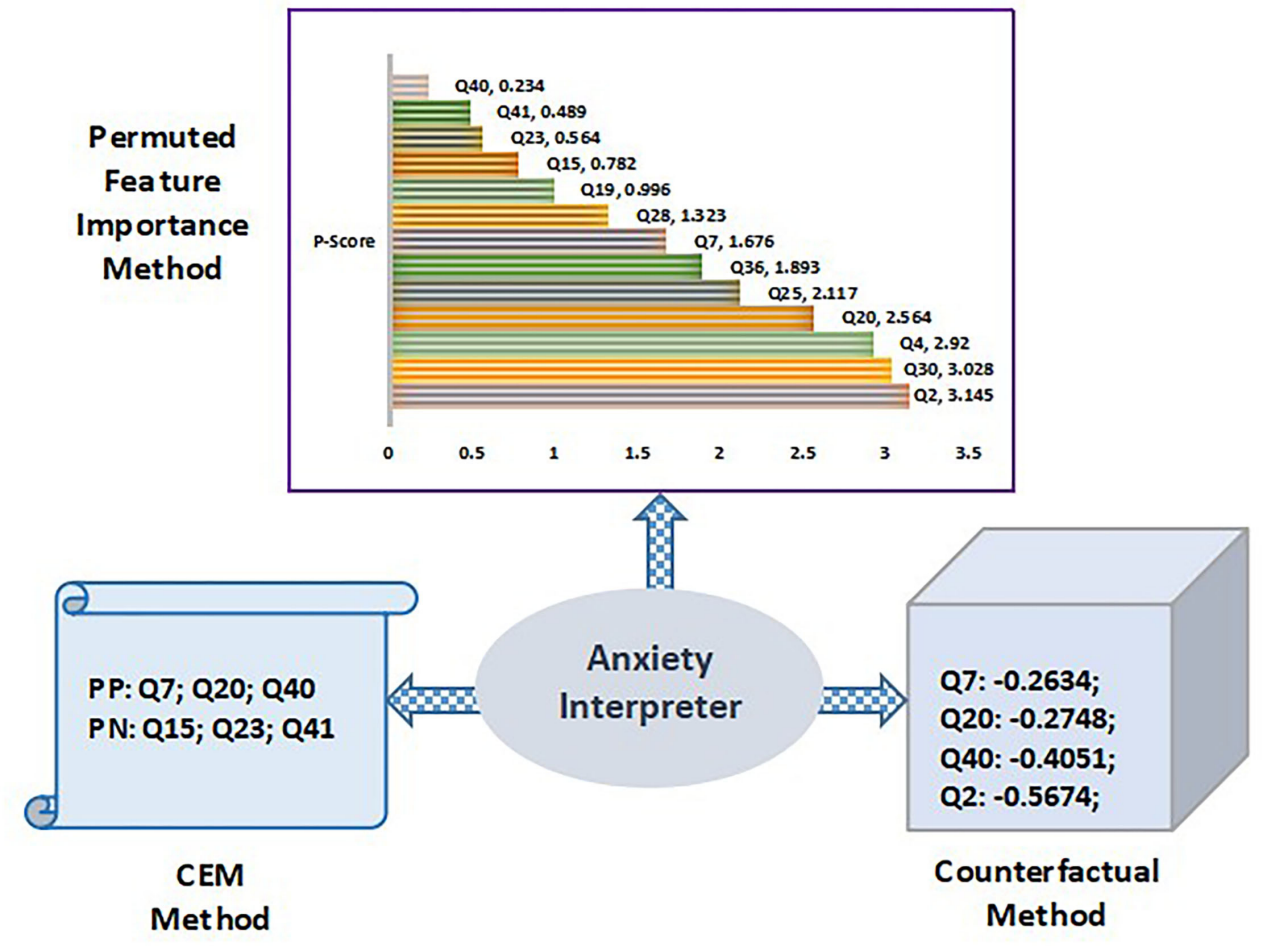
Illustration of anxiety interpreter outcomes.

Figure 13 highlights the test data of volunteer “V5” as the input to the Q-Prioritization phase to find the priority based relevant query set. This query set is applied to the proposed balanced decision tree model. The prediction outcome I presented was in the form of an interface. If any risk is detected then it will display the information based on the severity level. As it is observed that “V5” is detected with psychological risks in all three sets. An “MD” level of risk is recognized for both anxiety and depression disorder while an “M” risk level is detected with stress set. The predicted risk disorders are supported and regulated by a well-built interpreted engine. The interpreter engine explains the predicted outcome corresponding to all three disorders. Permuted feature importance method, CEM method, and Counterfactual method are the three different explanatory approaches implemented in this module for a comprehensive interpretation of predicted result class for each mental disorder set. Prediction interpretations for each set are presented in context to the detected outcome of volunteer “V5.”

### 5.3. Anxiety Interpreter

**Figure 14:**
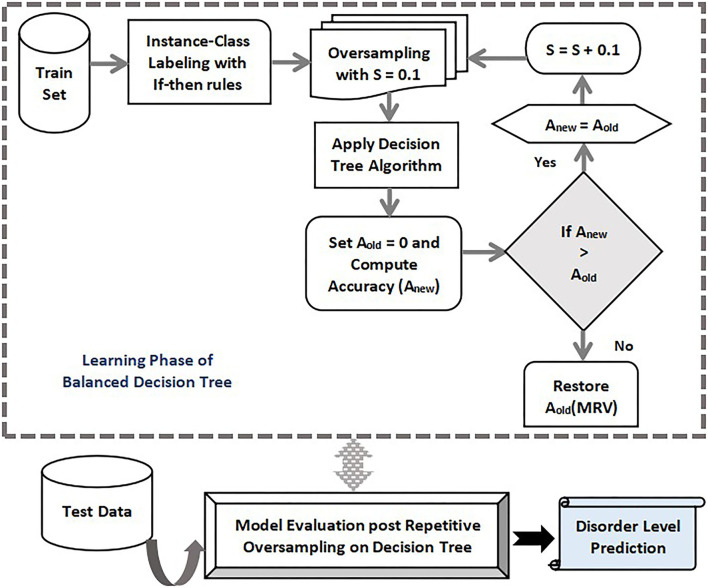
Training and testing phase with proposed balanced decision tree approach.

Anxiety interpreter acts as an reasoning interface that facilitates an effective explanation of the prediction outcome on the anxiety dataset which is depicted through the three significant methods discussed above. Figure 14 shows the anxiety interpreter.

In context to permuted feature importance method, the P-score value determines the importance of attributes. Q-score value changes with every iteration based on the individual position of attributes. Therefore, *P*-score also updates with every iteration. The value of *P*-score depends on the number of iterations and the accuracy value of all attributes in every round of execution. Figure 8 shows the ordered ranking of different queries based on their individual the P-score value for the volunteer “V5.” It is observed that “Q2” exhibit the highest importance score of 3.145 while “Q40” is the least important attribute with a score of 0.234. It shows the top ranked query attributes that impact the prediction decision making. CEM method is another approach to provide a suitable explanation for the prediction. It denotes the minimal queries to be present for the prediction of a specific class (PP) while simultaneously it also notifies the queries that should be absent during the prediction (PN). The inference of the CEM method is presented in tabular form in **Table 11**. It clearly indicates the scenario where queries Q7, Q20, and Q40 are the PP while Q15, Q23, and Q41 are regarded as PN. Based on the inference, relevant feedback is generated to the user for guidance as shown in Table 9.

**Table 9:** Feedback to anxiety interpreter with CEM method.

**Volunteer ID**	**Queries**	**PP**	**PN**	**Feedback (anxiety)**
V5	Q2; Q15; Q23; Q30; Q41	Q7; Q20; Q28; Q40	Q15; Q23; Q41	V5 is detected “Moderate” anxiety risk due to shakiness feeling, scared and panic. However, if he would have experienced faintness, difficulty in swallowing and trembling in hands then the risk would be upgraded to “Severe.”

The counterfactual method is the third interpreter approach used for effective explanation for prediction. As it can be seen that the original prediction for anxiety was “MD.” So to downgrade the prediction to “M,” the queries Q7, Q20, Q40, and Q2 represent the counterfactuals generated that are listed below. The negative values of the queries denote that the corresponding metric should be reduced. Thus, to reduce the anxiety risk to a mild level, “V5” should control the feeling of panic and constant shakiness. Furthermore, symptoms such as dryness in the mouth and calmness will assist in dealing with anxiety related issues.

Original predicted label: “MD”; Counterfactual prediction: “M”; Q7: –0.2634; Q20: –0.2748; Q40: –0.4051; Q2: –0.5674.

### 5.4. Stress Interpreter

**Figure 15:**
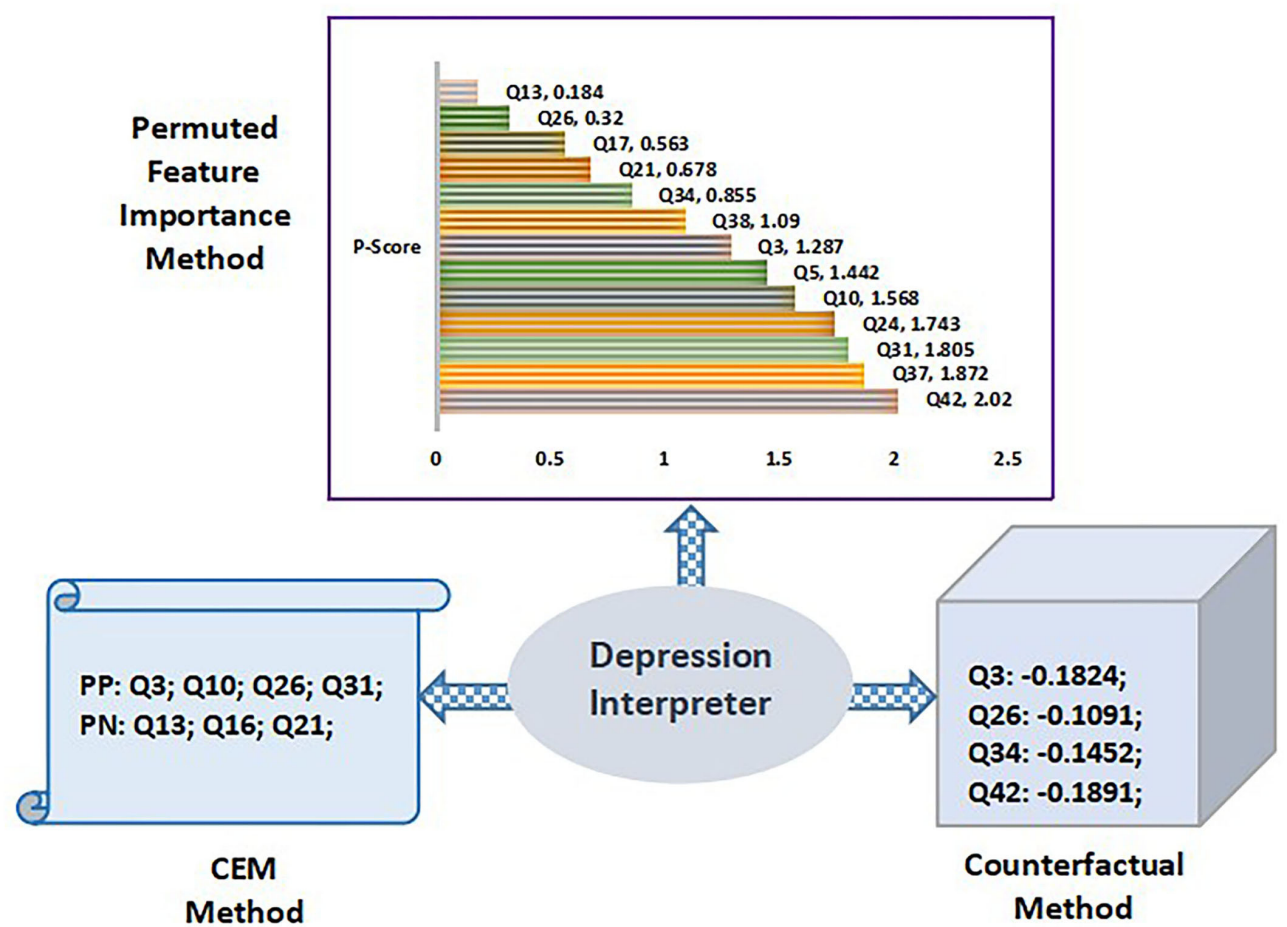
Illustration of depression interpreter outcomes.

Stress interpreter forms the second platform to provide an appropriate interpretation of the prediction outcome on the stress dataset. The explanation is shown through the same methods as highlighted above. Stress interpreter and its decision outcomes are highlighted in Figure 15.

According to the CEM method, the essential attribute queries (PP) recorded were Q1, Q22, Q29, and Q33 while the non-essential queries needed to be absent (PN) were Q6, Q12, Q14, and Q27. The stress feedback is shown in Table 10 where the relevant feedback is provided to the user based on the generated “PP” and “PN” values.

**Table 10:** Feedback to stress interpreter with CEM method.

**Volunteer ID**	**Queries**	**PP**	**PN**	**Feedback (stress)**
V5	Q1; Q6; Q11; Q12; Q22 Q27; Q32; Q33; Q39	Q8; Q1; Q14; Q22; Q29; Q35; Q33	Q6, Q12; Q14; Q27	V5 is detected with “Mild” stress risk due to being easily upset, unable to rest and maintain calm with excess tension. Moreover, if he tends to over-react more, get nervous and impatient with frequent irritation then he would develop “Moderate” risk.

The query importance is further validated with the counterfactual method to determine the attributes of the stress set that need to be regulated. The counterfactual results showed that “M” was the actual predicted class level, but it can be further regulated to “N” if certain attributes are properly taken care of. The attributes of impact detected were Q1, Q29, Q32, and Q33 as listed in Figure 2. So as per the counterfactual outcome, volunteer “V5” should avoid being frequently upset for silly reasons and should maintain calm. Furthermore, the usage of sensors and smart gadgets will enable remote monitoring of patients. Original predicted label: “M” Counterfactual prediction: “N” Q1: –0.2284; Q29: –0.1941; Q32: –0.3382; Q33: –0.1674.

### 5.5. Depression Interpreter

The user interface used in our work to provide an effective explanation for depression prediction is referred to as depression interpreter.

It also utilizes the same three approaches to provide a suitable explanation for prediction. Through permuted feature importance method, the priority ranking of all queries is obtained by their P-score value. The highest P-score of 2.02 is noted for query “Q42” while the least score of 0.184 is recorded for “Q13.” So it can be said that “Q42” is the most relevant query that had an impact on the prediction of depression in volunteer “V5.” The critical queries required for accurate prediction and queries that should be absent are computed using the CEM approach. It is noted that Q3, Q10, Q26, and Q31 are absolutely required for prediction of the same class level “MD” while queries “Q13,” “Q16,” and “Q21” should not impact the prediction process. On basis of the CEM method outcome, the feedback table is auto-generated as shown in Table 11.

**Table 11:** Feedback to depression interpreter with CEM method.

**Volunteer ID**	**Queries**	**PP**	**PN**	**Feedback (stress)**
V5	Q3; Q10; Q16; Q17; Q24 Q26; Q34; Q37; Q42	Q13; Q3; Q21; Q12; Q31; Q26; Q38; Q31	Q13; Q16; Q31	V5 is detected with “Moderate” depression risk due to high negative feeling, lack of objective in life, down-hearted and worthless mindset. However, if he continues to remain sad continuously with no zeal and worth in life feeling then the risk may be uplifted to “Severe.”

The query importance in depression risk prediction is evaluated with the counterfactual method to determine the queries that require attention. The counterfactual results showed that “MD” was the original predicted depression level which may be upgraded to “M” if four attributes are effectively controlled. These detected attributes were Q3, Q26, Q34, and Q42 as listed in Table 5. Hence, a volunteer “V5” should be more positive rather than being down-hearted. A volunteer should be kept busy to avoid anxiety related issues.

Q3: –0.1824; Q26: –0.1091; Q34: –0.1452; Q42: –0.1891;

The performance of the proposed model can be further enhanced in terms of speed and accuracy by taking image based datasets and using deep neural networks. Also, the entire system can be made smart by integrating sensors and actuators so that remote monitoring of patients can be possible. In the future, the model can be further upgraded to include more detailed questionnaires that capture the emotions of patients more accurately. Also, more mental risks types can be embedded in the research work to make the model more generic and broad.

## 6. Conclusion

Psychological health plays a crucial role in the well-being of a person. Any disturbance related to mental disorders may pose serious concerns in the future. Anxiety, stress, and depression contribute to the majority of psychological risks. Thus, it is important to recognize these health concerns and address them at the earliest. Many predictive learning based models are already being developed to predict these mental disorders. But most of these models simply predict the occurrence of such risks without providing any concrete explanation and reasoning. So reliability on these black-box models is questionable. So a more transparent and robust approach should be deployed such that the predictions of a model can be accurately visualized and interpreted. Thus, in our research, an explainable intelligence enabled predictive model is developed to not only accurately detect multiple severity levels of psychological health risks in an individual but also generate a transparent explanation of the contributing factors of a certain prediction. The model used a standard online questionnaire dataset to accumulate responses from online users. The data samples were filtered using a new method called Q-Prioritization through which the least priority queries in the dataset were dropped out. The query optimized dataset was trained with a new repetitive oversampled variant of the decision tree called the balanced decision tree method. After the successful learning phase, it was able to predict psychological issues such as anxiety, stress, and depression on a new test sample. Furthermore, a reasoning engine is used to visualize and interpret the prediction made by the model so as to determine the main contributing symptoms and less essential factors for a certain prediction of mental risks severity level. Permuted feature importance, contrastive explanation, and counterfactuals methods are utilized as constituents of the reasoning engine to empower the model with explainable intelligence. The deployed model gave very impressive results. The mean accuracy of prediction was 98.25% while the mean precision, recall, and F-score metric noted were 0.98, 0.977, and 0.979, respectively. It was observed that without applying Q-Prioritization, the mean accuracy significantly got reduced to 90.25%. The MSE generated with our model was a least of 0.026. Through the reasoning engine, the prediction outcome was more clear and transparent. Risk factors primarily responsible for a certain severity disorder level detection were quite evident and relatable. Based on the outcome, medical experts can suggest suitable recommendations to patients. Thus, the proposed multi-level psychological disorder predictive model driven by explainable intelligence can benefit medical professionals in the accurate diagnosis of patients concerned with psychological health risks. In the future, the model can be further upgraded to include more detailed questionnaires that capture the emotions of patients more accurately. Also, more mental risk types can be embedded in the research work to make the model more generic and broad.

## Data Availability Statement

The raw data supporting the conclusions of this article will be made available by the authors, without undue reservation.

## Author Contributions

SM, HT, HK, DG, KK, and SP: conceptualization and data collection, methodology, and manuscript drafting. KK: manuscript proofreading. All authors contributed to the article and approved the submitted version.

## Funding

This work was supported by the research work of Symbiosis Institute of Technology.

## Conflict of Interest

The authors declare that the research was conducted in the absence of any commercial or financial relationships that could be construed as a potential conflict of interest.

## Publisher's Note

All claims expressed in this article are solely those of the authors and do not necessarily represent those of their affiliated organizations, or those of the publisher, the editors and the reviewers. Any product that may be evaluated in this article, or claim that may be made by its manufacturer, is not guaranteed or endorsed by the publisher.
